# Hepatocyte Bcl-3 protects from death-receptor mediated apoptosis and subsequent acute liver failure

**DOI:** 10.1038/s41419-022-04946-y

**Published:** 2022-05-31

**Authors:** Nadine Gehrke, Marcus A. Wörns, Amrit Mann, Nadine Hövelmeyer, Ari Waisman, Beate K. Straub, Peter R. Galle, Jörn M. Schattenberg

**Affiliations:** 1 Department of Medicine, University Medical Center of the Johannes Gutenberg-University Mainz, Mainz, Germany; 2 Institute for Molecular Medicine, University Medical Center of the Johannes Gutenberg-University Mainz, Mainz, Germany; 3 Research Center for Immunotherapy, University Medical Center of the Johannes Gutenberg-University Mainz, Mainz, Germany; 4 Institute of Pathology, University Medical Center of the Johannes Gutenberg-University Mainz, Mainz, Germany; 5 Present Address: Department of Gastroenterology, Hematology, Oncology and Endocrinology, Klinikum Dortmund, Dortmund, Germany; 6 Present Address: Center for Thrombosis and Hemostasis, University Medical Center of the Johannes Gutenberg-University Mainz, Mainz, Germany

**Keywords:** Hepatotoxicity, Preclinical research

## Abstract

Acute liver failure (ALF) is a rare entity but exhibits a high mortality. The mechanisms underlying ALF are not completely understood. The present study explored the role of the hepatic B cell leukemia-3 (Bcl-3), a transcriptional regulator of nuclear factor-kappa B (NF-κB), in two independent models of ALF. We employed a recently developed transgenic mouse model in a C57BL6/J background comparing wild-type (WT) and transgenic littermates with hepatocyte-specific overexpression of *Bcl-3* (*Bcl-3*^Hep^) in the ALF model of d-galactosamine (d-GalN) and lipopolysaccharide (LPS). Additionally, the apoptosis-inducing CD95 (FAS/APO-1)-ligand was explored. *Bcl-3*^Hep^ mice exhibited a significant protection from ALF with decreased serum transaminases, decreased activation of the apoptotic caspases 8, 9, and 3, lower rates of oxidative stress, B-cell lymphoma 2 like 1 (BCL2L1/BCL-X_L_) degradation and accompanying mitochondrial cytochrome c release, and ultimately a decreased mortality rate from d-GalN/LPS compared to WT mice. d-GalN/LPS treatment resulted in a marked inflammatory cytokine release and stimulated the activation of signal transducer and activator of transcription (STAT) 3, c-Jun N-terminal kinases (JNK) and extracellular signal-regulated kinase (ERK) signaling comparably in the hepatic compartment of *Bcl-3*^Hep^ and WT mice. However, in contrast to the WT, *Bcl-3*^Hep^ mice showed a diminished rate of IkappaB kinase-beta (IKK-β) degradation, persistent receptor interacting protein kinase (RIPK) 1 function and thus prolonged cytoprotective nuclear factor-kappa B (NF-κB) p65 signaling through increased p65 stability and enhanced transcription. Likewise, *Bcl-3* overexpression in hepatocytes protected from ALF with massive hepatocyte apoptosis induced by the anti-FAS antibody Jo2. The protection was also linked to IKK-β stabilization. Overall, our study showed that Bcl-3 rendered hepatocytes more resistant to hepatotoxicity induced by d-GalN/LPS and FAS-ligand. Therefore, Bcl-3 appears to be a critical regulator of the dynamics in ALF through IKK-β.

## Introduction

Acute liver failure (ALF) is a rare but lethal entity. The treatment is based on withdrawal or suppression of the causing agent, but once injury and inflammation are initiated, hepatocellular injury is entertained by an intrinsic activation of cell death mechanisms that result in a fatal loss of liver tissue and function. Given that liver transplantation is the only definitive treatment option for these patients [1], there is high unmet need to identify the molecular mechanisms underlying the self-amplifying liver injury spiral, in order to develop biologically plausible treatments for patients with ALF.

Nuclear factor-kappa B (NF-κB) plays a central role in liver tissue homeostasis and regulating inflammation in response to various injurious challenges. In hepatocytes, NF-κB exerts primarily pro-survival effects by increasing the expression of genes encoding anti-apoptotic and anti-oxidant proteins, thus blocking cell death pathways [2–5]. Consequently, dysregulation of hepatocyte NF-κB has been linked to a variety of acute and chronic liver diseases [3–7]. NF-κB is assembled as homo- or heterodimer composed of the subunits p50/p105 (NF-κB1), p52/p100 (NF-κB2), p65 (RelA), c-Rel (Rel), or RelB and forms transcriptional complexes [8]. The primary mechanism regulating NF-κB transcriptional activity is through inhibitory kappa B (IκB) proteins including among others B cell leukemia-3 (Bcl-3) [9, 10].

Bcl-3 was originally identified as a proto-oncogene in leukemia [11] and is unique among the IκB proteins for nuclear localization. It contains two transactivation domains (TAD) that allow selective trans-activation or repression of NF-κB-dependent genes involving heterocomplex formation with p50 or p52 dimers [12, 13]. In addition, Bcl-3 has been reported to interact with other transcriptional regulators besides NF-κB [14–16]. Related to these pleiotropic functions of Bcl-3, we developed a transgenic mouse model overexpressing *Bcl-3* selectively in hepatocytes (*Bcl-3*^Hep^) to study its abundant role in liver disease [14, 17] and employed two established models of ALF.

## Results

### Bcl-3 protects from d-GalN/LPS-induced hepatotoxicity

**Fig. 1:**
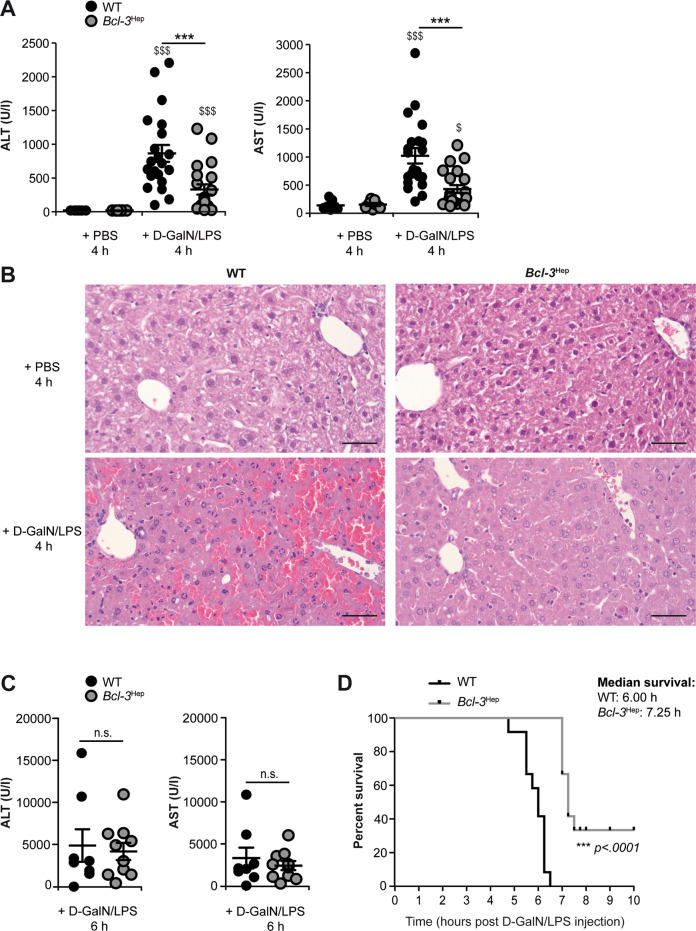
Conditional overexpression of *Bcl-3* in hepatocytes mitigated liver injury from d-GalN/LPS and promoted survival of mice. Liver injury following treatment with d-GalN/LPS was assessed by measurement of **A** serum alanine and aspartate aminotransferase (ALT and AST) levels, and **B** standard H&E staining of liver sections (scale bar: 50 µm, representative histological photomicrographs) in *Bcl-3*^Hep^ and WT mice at 4 h. **C** Serum transaminases in *Bcl-3*^Hep^ and WT mice at 6 h post d-GalN/LPS. **D** Kaplan–Meier survival plots for *Bcl-3*^Hep^ and WT mice after d-GalN/LPS injection. Data in **A** represent means ± SEM from four experiments with a total of *n* = 21 WT + d-GalN/LPS, *n* = 21 *Bcl-3*^Hep^ + d-GalN/LPS, *n* = 7 WT + PBS and n = 7 *Bcl-3*^Hep^ + PBS. Data in **C** represent means ± SEM of *n* = 10 mice/group, whereby 20% of the WT + d-GalN/LPS group was already dead at this time point. In **D** survival rate was monitored for 8 h after injection of d-GalN/LPS in a total of *n* = 12 WT and *n* = 10 *Bcl-3*^Hep^ mice. ***p < 0.001 for WT vs. *Bcl-3*^Hep^ and ^$^*p* < 0.05, ^$$$^*p* < 0.001 for PBS vs. d-GalN/LPS using Mann–Whitney *U* test (**A** and **C**) or a log-rank test (**D**). In **C** there was no statistical difference between the two groups in respect of the parameters.

d-GalN/LPS induced a rapid hepatic and systemic inflammatory response in *Bcl-3*^Hep^ and wild-type (WT) mice at 4 h characterized by inflammatory cytokines including tumor necrosis factor-α (TNF), interleukin (IL)-6, and IL-1α/β, and activation of hepatic signal transducer and activator of transcription 3 (STAT3) (Supplementary Fig. 1A–D). This was comparable between genotypes. Likewise, liver enzymes increased significantly (Fig. 1A, C), however this liver injury pattern was significantly attenuated in *Bcl-3*^Hep^ mice (Fig. 1A). Blinded histopathological examination of hematoxylin & eosin (H&E)-stained liver sections showed extensive areas of hepatocyte cell death from d-GalN/LPS, that was substantially reduced in *Bcl-3*^Hep^ mice (Fig. 1B). At 6 h post insult hepatic injury was comparable between the two genotypes. Still 20% of the WT mice succumbed to ALF at 6 h (Fig. 1C), while 20% of *Bcl-3*^Hep^ mice survived the insult beyond 8 h (Fig. 1D). Taken together, these data suggest that *Bcl-3* overexpression slowed the onset of ALF in a TNF-dependent model.

### Reduction in hepatocellular apoptosis in Bcl-3^Hep^ mice

**Fig. 2:**
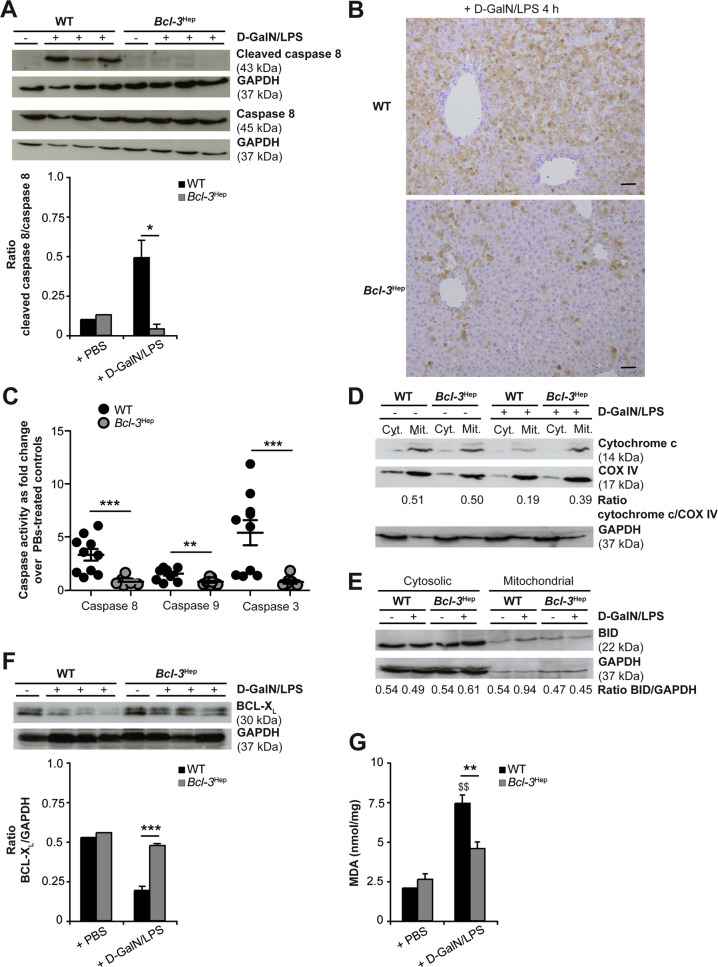
Hepatoprotection in *Bcl-3*^Hep^ mice against d-GalN/LPS is associated with a reduction of caspase activation, BCL-X_L_ degradation and mitochondrial ROS formation. Liver tissue of d-GalN/LPS-challenged *Bcl-3*^Hep^ and WT mice was harvested after 4 h for immunodetection of **A** activated caspase 8 by immunoblotting, **B** activated caspase 3 by immunhistochemical staining, and **C** activated caspase 8, 9, and 3 by caspase enzyme assays (means of *n* = 10 mice/group ± SEM). Activation of the mitochondrial apoptotic pathway was further evaluated by immunoblotting of **D** cytochrome c and **E** BID in cytosolic (cyt.) and mitochondrial (mit.) protein fractions, **F** BCL-X_L_ in whole liver tissue lysates, and **G** determination of MDA content (means of *n* = 10 WT + d-GalN/LPS, *n* = 6 *Bcl-3*^Hep^ + d-GalN/LPS, *n* = 2 PBS-treated controls per genotype ± SEM). In **A** and **D**–**F** representative immunoblots with densitometric analysis are shown. GAPDH and COX IV served as protein loading controls. In **B** representative histological photomicrographs (scale bar: 2000 µM) are depicted. *p < 0.05, **p < 0.01, ***p < 0.001 for WT vs. *Bcl-3*^Hep^ and ^$$^ *p* < 0.01 for PBS vs. d-GalN/LPS using unpaired, two-tailed Student’s *t*-test (**A**, **F**, and **G**) or Mann–Whitney *U* test (**C**).

Next, effector molecules involved in TNF cell death signaling were assessed. Activation of caspase 8 and 3 was significantly lower in *Bcl-3*^Hep^ mice at 4 h. Both truncated caspase fragments (Fig. 2A, B) as well as caspase activity assays (Fig. 2C) indicated reduced activation in *Bcl-3*^Hep^ mice. Likewise, enzymatic activity of caspase 9 was only detectable in the WT (Fig. 2C). Caspase 9 activation involves the mitochondrial cell death pathway following cleavage of BH3 interaction domain death agonist (BID) and the subsequent release of mitochondrial cytochrome c. Cytochrome c reduction was detectable in the mitochondrial fractions of WT mice, but not accompanied by increasing cytochrome c in the cytosolic fraction (Fig. 2D). In parallel, translocation of full-length BID protein from the cytosol to the mitochondria fraction was detectable only in the WT 4 h after d-GalN/LPS, but not accompanied by BID cleavage (Fig. 2E). Also, increased pro-apoptotic BCL-2-associated X protein (BAX) was detectable at 4 h in both genotypes (Supplementary Fig. 2A) and BCL-2 antagonist/killer (BAK) expression was unchanged (Supplementary Fig. 2B).

To address the mechanism by which hepatocellular Bcl-3 protects from ALF, we investigated gene expression of key regulators of apoptosis including cellular inhibitor of apoptosis protein (cIAP)-1, X-linked inhibitor of apoptosis (XIAP), the anti-apoptotic BCL-2 family members B-cell lymphoma 2 (BCL-2), B-cell lymphoma 2 like 1 (BCL2L1/BCL-X_L_), and myeloid cell leukemia-1 (MCL-1), cellular FLICE (FADD-like IL-1β-converting enzyme)-inhibitory protein long/short (cFLIP_L_/cFLIP_S_), and A20 (Table 1A). All of these share the regulation through NF-κB but did not show differences at 4 h. The most striking differences between the genotypes were observed for BCL-X_L_, which was downregulated only in the WT from d-GalN/LPS (0.6-fold in WT vs. 1.7-fold in *Bcl-3*^Hep^, *p* = n.s.), and c-FLIP_S_, which was upregulated in *Bcl-3*^Hep^ livers, while unaffected in WT animals (1.0-fold in WT vs. 1.4-fold in *Bcl-3*^Hep^, *p* < 0.05). cFLIP_s_ protein levels were not detectable by immunoblotting (Supplementary Fig. 2C). Protein expression of BCL-X_L_ in WT was significantly lower compared to *Bcl-3*^Hep^ mice following d-GalN/LPS (Fig. 2F). Additionally, increasing malondialdehyde (MDA) suggestive of reactive oxygen species (ROS) were detectable in WT only (Fig. 2G). Levels of the anti-apoptotic XIAP protein did not follow this pattern and were increased in the WT early after d-GalN/LPS, and declined in *Bcl-3*^Hep^ mice (Supplementary Fig. 2D). In summary, *Bcl-3* overexpression suppresses TNF-induced hepatocyte apoptosis involving decreased caspase and mitochondrial cell death pathway activation, as well as lower rates of oxidative stress.

**Table 1:** Hepatic gene expression of (A) anti-apoptotic proteins and (B) NF-κB subunits in *Bcl-3*^Hep^ and WT mice at 4 h post d-GalN/LPS challenge and in control animals.

(A)				
	WT + PBS	WT + d-GalN/LPS	*Bcl-3*^Hep^ + PBS	*Bcl-3*^Hep^ + d-GalN/LPS
BCL-2	1.00 ± 0.21	41.46 ± 8.58 ^($)^	0.88 ± 0.08	23.39 ± 1.72 ^($$)^
BCL-X_L_	1.00 ± 0.12	0.59 ± 0.03 ^($)^	0.57 ± 0.02	0.95 ± 0.43
cIAP1	1.00 ± 0.11	4.10 ± 0.48 ^($)^	0.83 ± 0.10	3.00 ± 0.35 ^($$)^
c-FLIP_L_	1.00 ± 0.07	0.89 ± 0.05	0.92 ± 0.04	0.81 ± 0.03
c-FLIP_S_	1.00 ± 0.06	0.96 ± 0.06	0.78 ± 0.05	1.06 ± 0.06
MCL-1	1.00 ± 0.04	0.60 ± 0.08	1.17 ± 0.24	0.66 ± 0.12
A20	1.00 ± 0.06	5.41 ± 0.80 ^($)^	0.76 ± 0.06	4.29 ± 0.10 ^($)^
XIAP	1.00 ± 0.04	0.81 ± 0.03 ^($)^	0.95 ± 0.1	0.74 ± 0.06
**(B)**				
p50/p105	1.00 ± 0.09	2.44 ± 0.25 ^($)^	0.83 ± 0.05	1.90 ± 0.1 ^($$)^
c-Rel (Rel)	1.00 ± 0.17	1.03 ± 0.13	0.77 ± 0.06	1.70 ± 0.55 ^($)^
p65 (RelA)	1.00 ± 0.25	0.39 ± 0.13	0.19 ± 0.02 ^(^*^)^	0.53 ± 0.08 ^($)^
RelB	1.00 ± 0.08	2.84 ± 0.53 ^($)^	0.99 ± 0.13	2.62 ± 0.02 ^($)^

Data in (A) and (B) are shown as mean of *n* = 3-6 mice/group ± SEM. *p < 0.05 for WT vs. *Bcl-3*^Hep^ and ^$^*p* < 0.05, ^$$^*p* < 0.01 for PBS vs. d-GalN/LPS using Mann–Whitney *U* test (A and B). There was no statistically significant difference between WT + d-GalN/LPS vs. Bcl-3Hep + d-GalN/LPS in respect of the parameters.

### Bcl-3 stabilizes hepatic NF-κB p65

**Fig. 3:**
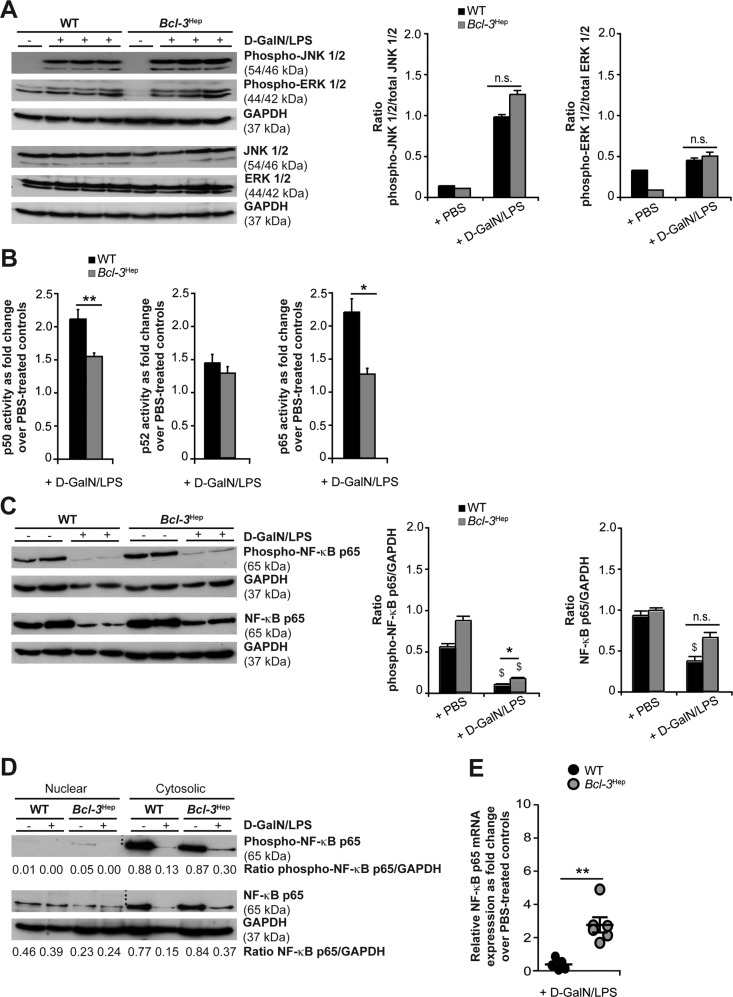
Activation of hepatic JNK, ERK and NF-κB in *Bcl-3*^Hep^ and WT mice in response to d-GalN/LPS. **A** Immunodetection of phospho-JNK (Thr183/Tyr185), phospho-ERK (Thr202/Tyr204) and total JNK/ERK protein in whole liver tissue lysates from *Bcl-3*^Hep^ and WT mice at 4 h post d-GalN/LPS challenge. **B** NF-κB p65, p50 and p52 activity was quantified by a functional binding assay. Ratio of relative NF-κB p65/p50/p52 activity in d-GalN/LPS-challenged *Bcl-3*^Hep^ vs. WT mice is shown (means of *n* = 7 WT + d-GalN/LPS, *n* = 8 *Bcl-3*^Hep^ + d-GalN/LPS and *n* = 7 PBS-treated controls per genotype ± SEM). **C** Immunoblotting of phospho-NF-κB p65 (Ser536) and total NF-κB p65 in whole liver tissue lysates and **D** after cytosolic vs. nuclear cell fractionation. **E** Relative hepatic NF-κB p65 gene expression in d-GalN/LPS-challenged *Bcl-3*^Hep^ vs. WT mice as fold change over PBS-treated controls (means of *n* = 6 mice per genotype ± SEM). In **A**, **C** and **D** representative immunoblots with densitometric analysis are shown. *p < 0.05, **p < 0.01 for WT vs. *Bcl-3*^Hep^ and ^$^*p* < 0.05 for PBS vs. d-GalN/LPS using unpaired, two-tailed Student’s *t*-test (**A**–**C**) or Mann–Whitney *U* test (**E**).

In hepatocytes, TNF-induced apoptosis is regulated by mitogen-activated protein kinases (MAPK) and NF-κB signaling pathways. Phosphorylation of both c-Jun N-terminal kinases (JNK) and extracellular signal-regulated kinase (ERK) increased irrespective of the genotype at 4 h after d-GalN/LPS (Fig. 3A). Coincident with MAPK activation, increased NF-κB p50, p52, and p65 DNA-binding activity were detected in whole liver lysates following d-GalN/LPS treatment (Fig. 3B). Differences between the two genotypes related to increased activity of p50 and p65 in the WT compared to *Bcl-3*^Hep^ mice (p50: 2.1-fold in WT vs. 1.5-fold in *Bcl-3*^Hep^, *p* < 0.01, p65: 2.2-fold in WT vs. 1.3-fold in *Bcl-3*^Hep^, *p* < 0.05), pointing to a stronger activation of the classical (or canonical) NF-κB pathway. To explore the role of cytoprotective p65 NF-κB, we visualized p65 expression by confocal microscopy. At baseline, the p65 signal was predominantly located in the cytosol of hepatocytes, translocating to the nuclear compartment upon d-GalN/LPS treatment (Supplementary Fig. 3). Compared to the WT, *Bcl-3*^Hep^ mice displayed reduced basal levels as well as translocation of NF-κB p65 during ALF.

Post-translational modifications including phosphorylation at serine 536 (Ser536) are required to increase p65 transcriptional activity. Ser536 phosphorylation will lead to nuclear localization of p65 and enhance its binding to coactivators and transcription factors [18]. Immunoblots of liver tissue lysates demonstrated significant lower expression of total and phospho-Ser536 p65 protein - in particular in WT mice at 4 h of D-GalN/LPS (Fig. 3C). We observed significant different phospho-Ser536 p65 levels between WT and *Bcl-3*^Hep^ mice following d-GalN/LPS (*p* < 0.05). Cell fractionation and separate analysis of cytosolic and nuclear extracts confirmed a reduction of the cytosolic amounts of both total and phospho-Ser536 p65 protein in the WT group that was 2-fold stronger compared to the *Bcl-3*^Hep^ group (Fig. 3D). These changes were attributable to degradation rather than increased nuclear translocation in response to d-GalN/LPS. On contrast, a relatively stable, albeit lower, expression of total NF-κB p65 was observed in the nuclear fraction of *Bcl-3*^Hep^ mice compared to those of WT mice (Fig. 3D). In parallel, we did not observe Ser536-phosphorylated p65 in the nuclear fractions at 4 h, suggesting that the Bcl-3’s effects did not depend on S536-phosphorylation status of nuclear p65 (Fig. 3D). In contrast to the WT, *Bcl-3*^Hep^ mice displayed a relatively low basal mRNA expression level of p65 (*p* < 0.05, Table 1B), but exhibited increasing p65 mRNA expression following D-GalN/LPS (0.4-fold in WT vs. 2.8-fold in *Bcl-3*^Hep^, *p* < 0.01, Fig. 3E), reflecting an increased stability of the transcript resp. an increase in gene transcription. Levels of NF-κB p50 and RelB did not significantly differ between the genotypes. The Rel family member c-Rel is involved in liver regeneration and was significantly upregulated in the hepatic compartment of *Bcl-3*^Hep^ mice 4 h after d-GalN/LPS (Table 1B).

### Cell death in Bcl-3^Hep^ hepatocytes from ActD/TNF is depending on caspase, but not JNK activation

**Fig. 4:**
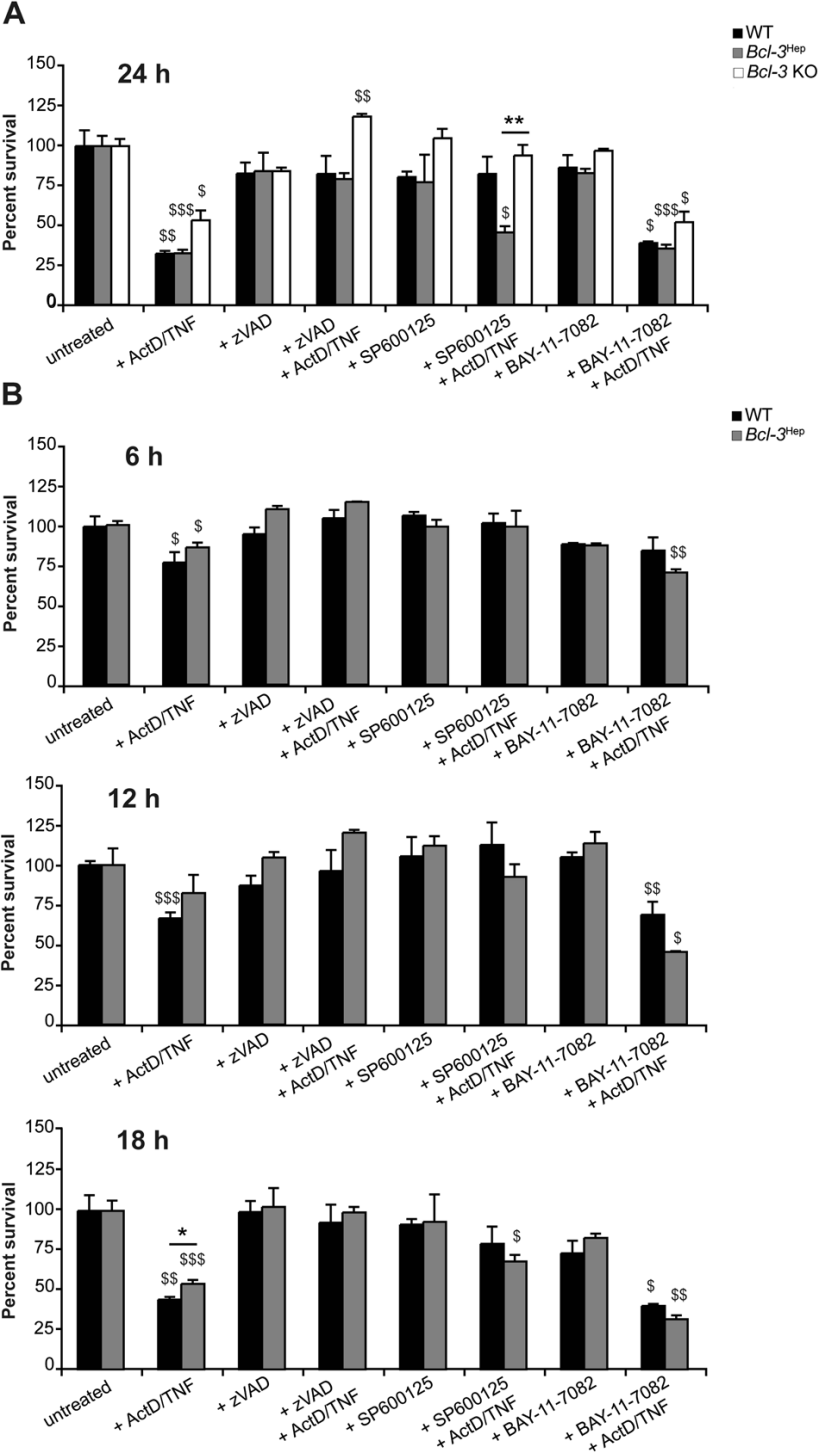
Rate of TNF-induced hepatocyte cell death in vitro. **A** Primary hepatocytes derived from *Bcl-3*^Hep^, WT, and *Bcl-3* KO mice were treated ex vivo with ActD (200 ng/mL) and TNF (10 ng/mL) to induce TNF-R driven cell death. Pan caspase inhibitor zVAD (50 µM), JNK inhibitor SP600125 (100 µM), or IKK inhibitor BAY-11-7082 (10 µM), where ever indicated, were added 1 h before ActD/TNF-treatment to examine the activity of caspases, JNK and NF-κB signaling in TNF-induced cell death in *Bcl-3*^Hep^, WT, and *Bcl-3* KO hepatocytes. After 24 h cell viability was assessed by MTT colorimetric assay relative to untreated samples. **B** Time course analysis of ActD/TNF-induced cell death in *Bcl-3*^Hep^ and WT hepatocytes determined by MTT assays after 6, 12, and 18 h. Numerical data in mean ± SEM of **A** three or **B** two independent experiments performed at least in duplicate readings. *p < 0.05, **p < 0.01 for WT vs. *Bcl-3*^Hep^ or *Bcl-3*^Hep^ vs. *Bcl-3* KO and ^$^*p* < 0.05, ^$$^*p* < 0.01, ^$$$^*p* < 0.001 for untreated vs. treated hepatocytes according to an unpaired, two-tailed Student’s *t*-test (**A** and **B**).

In order to explore the mechanisms related to the anti-apoptotic effect observed with Bcl-3, we employed small molecular inhibitors ex vivo. Primary hepatocytes isolated from *Bcl-3*^Hep^ mice were treated with TNF and the transcriptional inhibitor actinomycin D (ActD). Caspase-dependent cell death was comparable in both genotypes at 24 h (Fig. 4A) and responsive to caspase inhibition by zVAD. Remarkably, while JNK inhibition using SP600125 completely prevented cell death from ActD/TNF in WT hepatocytes, the inhibitory effect of SP600125 was less potent in *Bcl-3*^Hep^ hepatocytes. In *Bcl-3* knockout (KO) hepatocytes, treatment with SP600125 or zVAD was able to efficiently abrogate cell death from ActD/TNF (Fig. 4A). Blockade of NF-κB using the IkappaB kinase (IKK) inhibitor BAY-11-7082 had no impact on ActD/TNF-induced cell death in cultured *Bcl-3*^Hep^, WT, or *Bcl-3* KO hepatocytes at 24 h. A time course analysis of ActD/TNF-induced cell death in *Bcl-3*^Hep^ hepatocytes, however, showed a delay compared to WT cells (Cell death in WT vs. *Bcl-3*^Hep^ hepatocyte cultures: 24.1% vs. 14.7% at 6 h, 33.3% vs. 17.6% at 12 h, and 56.0% vs. 46.0% at 18 h, *p* < 0.05, Fig. 4B). ActD/TNF-induced cell death ex vivo was prevented by zVAD, but not by SP600125. Remarkably, the protective effect of *Bcl-3* overexpression in primary hepatocytes was lost by blocking the IKK/NF-κB pathway with BAY-11-7082.

### IKK-β protects Bcl-3 overexpressing hepatocytes from apoptosis in vivo

**Fig. 5:**
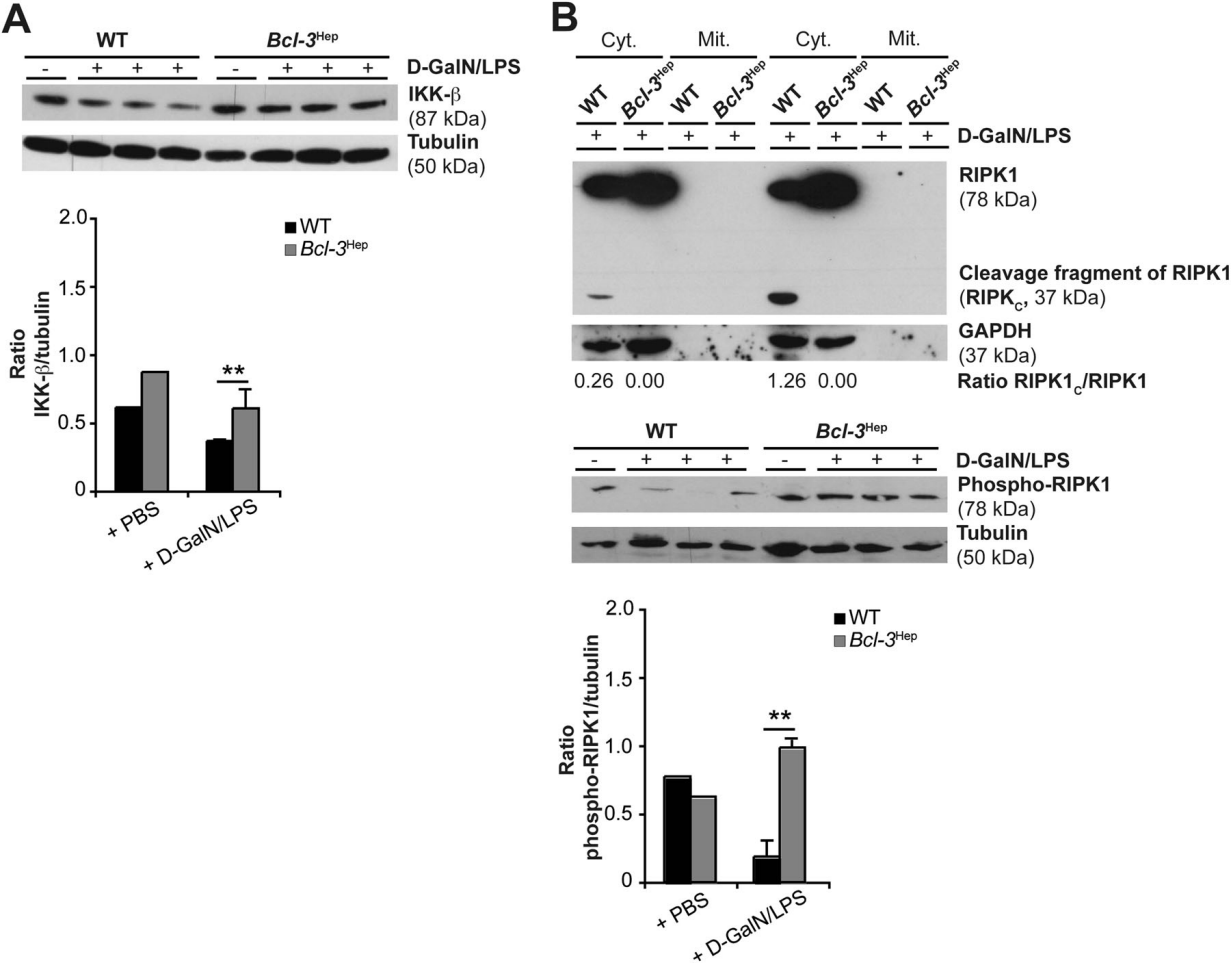
Reduction of hepatic IKK-β, cleavage and Ser166-dephosphorylation of RIPK1 during apoptosis induced by d-GalN/LPS does not occur in *Bcl-3*^Hep^ mice. **A** Immunoblotting was performed with a specific antibody against the C terminus of IKK-β in whole liver tissue lysates from *Bcl-3*^Hep^ and WT mice at 4 h after d-GalN/LPS challenge and corresponding controls. **B** To test the involvement of RIPK1 in d-GalN/LPS-induced liver injury, RIPK1 protein levels were analyzed in cytosolic (cyt.) and mitochondrial (mit.) protein fractions from two representative *Bcl-3*^Hep^ and WT mice treated with d-GalN/LPS. Phospho-RIPK1 (Ser166) levels were determined in whole liver homogenates from *Bcl-3*^Hep^ and WT mice at 4 h post D-GalN/LPS or PBS injection. In **A** and **B** representative western blots with densitometric analysis are shown. Tubulin and GAPDH served as protein loading controls. **p < 0.01 for WT vs. *Bcl-3*^Hep^ according to an unpaired, two-tailed Student’s *t*-test (**A** and **B**).

The regenerative response in primary hepatocytes is largely dependent on activation of IKK-β (also called IKK2), which is required for the TNF-induced pro-survival transcriptional activity of the NF-κB subunit RelA/p65 and also protects against apoptosis through a NF-κB-independent axis [19–21]. Hence, we examined IKK-β expression in d-GalN/LPS-induced acute liver injury. Immunoblotting showed significantly lower IKK-β in WT liver tissue at 4 h post d-GalN/LPS compared to *Bcl-3*^Hep^ (Fig. 5A). Thus, this data supports that *Bcl-3* overexpression stabilizes IKK-β during TNF-induced apoptosis.

IKK kinases maintain receptor interacting protein kinase 1 (RIPK1) in a pro-survival mode during TNF signaling, while their inhibition can sensitize cells to death through RIPK1-dependent apoptosis or necroptosis via RIPK3 [21, 22]. No significant changes in RIPK1 or RIPK3 gene expression levels were seen (Supplementary Table 1A). RIPK1 protein was cleaved in the liver cytosolic fractions from d-GalN/LPS-challenged WT mice, whereas no RIPK1 degradation occurred in *Bcl-3*^Hep^ mice (Fig. 5B). These data are aligned with a role of caspase-dependent RIPK1 inhibition of pro-survival IKK/NF-κB signaling [23]. Dephosphorylation of RIPK1 at Ser166 in WT livers in response to d-GalN/LPS (Fig. 5B) pointed to an inactivation of RIPK1 enzyme activity [24, 25]. By contrast, *Bcl-3*^Hep^ mice showed relative stable phospho-Ser166 RIPK1 protein levels at a level above the controls. The use of a small molecule inhibitor of RIPK1 kinase activity necrostatin (Nec-1) aggravated liver injury from d-GalN/LPS in both genotypes (Supplementary Table 1B). This was paralleled by a significant induction of RIPK1 and RIPK3 transcripts (Supplementary Table 1A). These data suggest that inhibition of RIPK1 kinase activity with Nec-1 is detrimental in this model in line with previous observations across different models and cell types [26–28], and support that stable RIPK1 functioning in *Bcl-3*^Hep^ mice in response to TNF is protective for hepatocyte survival.

### Hepatocyte-specific Bcl-3 overexpression protects mice from FAS-mediated hepatocyte apoptosis

**Fig. 6:**
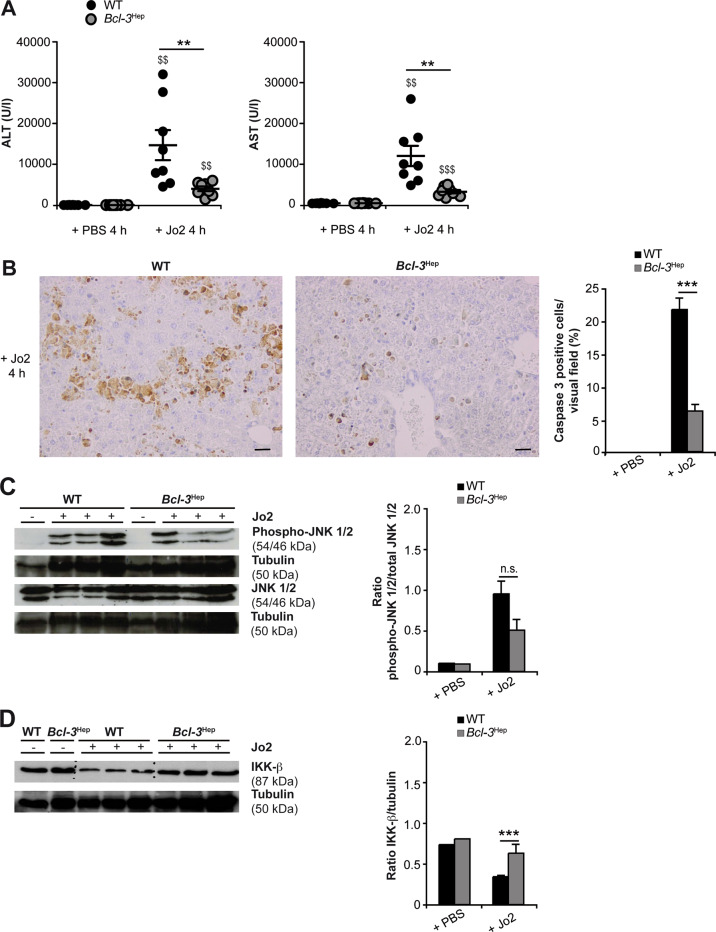
Agonistic anti-FAS (CD95/APO-1) antibody Jo2-induced acute liver injury is attenuated in *Bcl-3*^Hep^ mice. Liver injury was assessed by measurement of **A** serum ALT and AST levels and **B** caspase 3 activity on liver histology at 4 h post Jo2. **C** Hepatic JNK activity and **D** IKK-β expression were analyzed by immunoblotting in whole liver tissue lysates. Data in **A** represent means ± SEM from two experiments with a total of *n* = 8 WT + Jo2, *n* = 8 *Bcl-3*^Hep^ + Jo2, *n* = 7 WT + PBS, and *n* = 7 *Bcl-3*^Hep^ + PBS. In **B** representative histological photomicrographs (scale bar: 2000 µM) are depicted. In **C** and **D** representative western blots with densitometric analysis are shown. **p < 0.01, ***p < 0.001 for WT vs. *Bcl-3*^Hep^ and ^$$^*p* < 0.01, ^$$$^*p* < 0.001 for PBS vs. Jo2 using Mann–Whitney *U* test (**A**: ALT) or unpaired, two-tailed Student’s *t*-test (**A**: AST and **B**–**D**). In **C** there was no statistical difference between the two groups in respect of JNK activation.

**Fig. 7:**
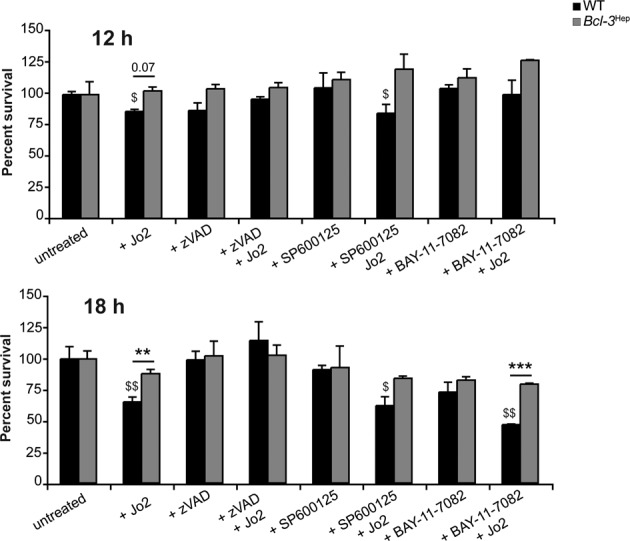
Rate of Jo2-induced hepatocyte cell death in vitro. Primary *Bcl-3*^Hep^ and WT hepatocytes were treated ex vivo with Jo2 (50 ng/mL). Pan caspase inhibitor zVAD (50 µM), JNK inhibitor SP600125 (100 µM), or IKK inhibitor BAY-11-7082 (10 µM), where ever indicated, were added 1 h before Jo2-treatment. After 12 and 18 h cell survival was assessed by MTT assay. Untreated hepatocytes were used as baseline controls. Numerical data in mean ± SEM of two independent experiments performed at least in duplicate readings. **p < 0.01, ***p < 0.001 for WT vs. *Bcl-3*^Hep^ and ^$^*p* < 0.05, ^$$^*p* < 0.01 for untreated vs. treated hepatocytes according to an unpaired, two-tailed Student’s *t*-test.

Finally, we employed a second model of death-receptor mediated hepatocellular injury and ALF. Administration of the murine CD95 (FAS/APO-1)-stimulating antibody Jo2 resulted in massive liver cell apoptosis in WT mice accompanied by ALT and AST enzymes and a significant increase in caspase 3-positive apoptotic hepatocytes at 4 h. Interestingly, liver injury in *Bcl-3*^Hep^ mice from Jo2 at this time point was minimal (Fig. 6A, B). FAS stimulation by Jo2 activated the hepatic JNK pathway, but there was no significant difference between *Bcl-3*^Hep^ and WT mice (Fig. 6C and Supplementary Fig. 4), indicating that JNK activation was not required for FAS-mediated caspase activation and liver injury [29, 30]. Previous studies have suggested that FAS stimulation does not activate NF-κB in hepatocytes, while inhibition of NF-κB promotes apoptosis induced through CD95 [31]. FAS-induced apoptosis also requires proteolysis of IKK-β. Remarkably, in response to Jo2, IKK-β was clearly reduced in WT liver tissue at 4 h, whereas hepatic IKK-β expression remained unaffected in *Bcl-3*^Hep^ mice (Fig. 6D). This suggests that *Bcl-3* overexpression is linked to persistence of IKK-β activity over time in this model. Ex vivo stimulation of primary *Bcl-3*^Hep^ and WT hepatocytes recapitulated lower levels of Jo2-induced caspase-dependent apoptosis in *Bcl-3*^Hep^-derived hepatocytes (Cell death in WT vs. *Bcl-3*^Hep^ hepatocyte cultures: 13.6% vs. 0% at 12 h, *p* = 0.07, and 34.8% vs. 11.9% at 18 h, *p* < 0.01), which was independent of JNK (SP600125) and NF-κB (BAY-11-7082) inhibition (Fig. 7). By contrast, the hepatoxic effects of Jo2 in WT hepatocytes were augmented by NF-κB inhibition.

## Discussion

In order to develop novel therapeutic interventions in ALF, the molecular mechanisms underlying hepatocyte survival or death need to be clearly understood. Bcl-3 has been identified as a key survival factor for various cell types, but also as an anti-inflammatory regulator of immune cells related to its regulatory function on NF*-*κB activity. We and others have previously shown that Bcl-3 interacts also with non-NF-κB proteins and exerts important effects on cell homeostasis and metabolism [14, 16, 32]. The current analysis explored mice with a hepatocyte-specific overexpression of *Bcl-3* in two models of experimental ALF in vivo and in a hepatocyte-based ex vivo culture system. Our results indicate that *Bcl-3* overexpression in hepatocytes reduced the sensitivity towards TNF-R and FAS-mediated apoptosis in vivo and ex vivo resulting in different dynamics of cell death with protection from Bcl-3 and increased overall survival. This is in contrast to a recently published report from Hu and colleagues, which employed global *Bcl-3* KO mice in the TNF/D-GalN model and the hepatic cell lines HepG2 and LO2 for in vitro analyses [33].

Here, we show that Bcl-3 acts as a pro-survival factor in hepatocytes by a mechanism located upstream of the mitochondrial cell death pathway and the activation of caspases. In fact, only WT mice showed a clear reduction of BCL-X_L_ protein, leading to the loss of mitochondrial integrity through a decrease in mitochondrial membrane potential and ROS production [34] and through BAD, truncated (t)BID and BAX [35]. Likewise, enhanced oxidative stress as measured by increasing MDA levels was evident in livers of d-GalN/LPS-challenged WT mice compared to *Bcl-3*^Hep^ mice. This was accompanied by caspase 8 activation, translocation of the pro-apoptotic BID protein to mitochondria, cytochrome c release and activation of the downstream caspases 9 and 3 in the WT. Interestingly, we did not detect proteolytic cleavage of BID, which has been indicated as a trigger of cytochrome c release [36]. Kinetics of BID cleavage and very low levels of tBID at 4 h post d-GalN/LPS could be related to this. On the other hand, full-length BID was also described to exhibit apoptotic and cytochrome c releasing activity [37]. In neuronal cells even in the absence of detectable BID cleavage, the MAPK JNK phosphorylated full-length BID, which as a consequence translocated to mitochondria and potentiated extrinsic TNF-induced apoptosis signaling [38]. In addition, non-BID-mediated mechanisms of cytochrome c release have been described [39]. Further expression analyses of proteins involved in the mitochondrial apoptosis pathway, however, revealed that Bcl-3 did not influence the levels of pro-apoptotic BAX, BAK, BCL-2, or MCL1, nor the anti-apoptotic cIAP1, c-FLIP or A20 proteins.

Sustained activation of JNK plays a critical role in hepatoxicity by d-GalN/LPS and ROS [40, 41]. Remarkably, both genotypes exhibited comparable early hepatic JNK activation, suggesting a JNK-independent effect. Supporting this, in vitro studies in primary hepatocytes indicated that cell death from ActD/TNF-treatment could be prevented by JNK inhibition only in WT hepatocytes. We can only speculate on the role of Bcl-3 counteracting the adverse effects of JNK signaling and switching TNF signaling to a protective response. One potential link relates to the activating protein-1 (AP-1) complex, which is transcriptionally regulated by Bcl-3 [42]. In hepatocytes, c-Jun/ AP-1 functionally antagonizes the cell death-promoting functions of JNK [43]. In previous studies using RNA-seq we identified JunB to be differentially expressed in *Bcl-3*^Hep^ mice [14]. JunB is able to inhibit the transcriptional activity of c-Jun [44] and was significantly downregulated in *Bcl-3*^Hep^ liver tissue (*p* < 0.01, hit: 456) [14]. Thus, transcriptional activity of c-Jun or other members of the AP-1 family might exert a protective signal through *Bcl-3* overexpression.

Activation of NF-κB p65 antagonizes hepatocyte cell death induced by TNF, by increasing the expression of genes encoding anti-apoptotic and anti-oxidant proteins, which block the activity of either death receptors or the mitochondrial pathway of apoptosis, and additionally by repression of JNK activity [2]. While *Bcl-3*^Hep^ livers exhibited reduced NF-κB 65 and p50 DNA-binding activities and p65 translocation in response to d-GalN/LPS, WT mice showed a stronger degradation of total and Ser536-phosphorylated NF-κB p65 protein - in particular in the cytosol - after d-GalN/LPS and no upregulation of p65 gene expression.

NF-κB is regulated by multiple mechanisms. Phosphorylation of p65 at Ser536 by IKK-β promotes the nuclear translocation and its transcription activity, but also accelerates its ubiquitination and proteasomal degradation [45] - a process that is involved in terminating the NF-κB protection during TNF signaling. Given that IKK-β is the predominant kinase to phosphorylate NF-κB p65 in its TAD in hepatocytes [46], the relatively stable IKK-β expression in *Bcl-3*^Hep^ mice compared to the WT indicates that this could be a crucial difference. At this point it remains undefined if stabilization of IKK-β is an effect of *Bcl-3* overexpression or depends on caspase 3-related caspases as previously observed [47].

Deletion or inactivation of IKK-β increases the sensitivity of rat hepatocytes [48] towards TNF-induced apoptosis. In a hepatocyte-specific *Ikk*2 knockout mouse model (*Ikkβ*^Δhep^), Maeda and colleagues demonstrated - in agreement with our data - that lack of functional IKK-β in hepatocytes resulted in massive hepatocyte apoptosis and higher lethality in mice given d-GalN/LPS - likely through substantial inhibition of survival gene expression [4]. Interestingly, *Ikkβ*^Δhep^ mice were also highly susceptible to liver injury following ConA-induced activation of T cells, that express cell-bound TNF, through induction of prolonged JNK activation [4]. Also, soluble TNF induced substantial apoptosis in *Ikkβ*^Δhep^ hepatocytes, accompanied by sustained JNK activation [4]. These findings indicate that the anti-apoptotic function of IKK-β might be related to cell-bound and/or high levels of circulating TNF and is partially dependent on JNK activity. Given the comparable levels of circulating TNF in both genotypes, one hypothesis on the slower emergence of liver injury in *Bcl-3*^Hep^ is related to IKK-β. This is supported by the observation that Bcl-3 exerted a strong protective effect against FAS-induced hepatocyte apoptosis and liver injury, which was linked to IKK-β stabilization. The hepatoprotective function of IKK-β against FAS-mediated apoptosis has previously been shown [49].

IKK-α and IKK-β - in addition to their known function in NF-κB activation - can also directly phosphorylate RIPK1 at distinct regions, thereby regulating cell viability independent of NF-κB [22]. Any regulator of IKK-α/IKK-β activation results in defective phosphorylation of RIPK1 in complex I leading to pro-death complex IIb assembly and RIPK1 kinase-dependent necroptosis [50]. Furthermore, this is also observed when TNF-induced transcription is inhibited and RIPK1 is de-ubiquitinated by enzymes such as cylindromatosis (CYLD). Interestingly, CYLD was recently identified as a physical interaction partner of Bcl-3 [33, 51]. In the current analysis, we observed that cleavage of RIPK1 occurred in parallel to lower levels of Ser166 posphorylation of RIPK1 in the WT. This suggests that caspase-dependent RIPK1 cleavage shuts down protective IKK/NF-κB signaling pathways. It remains to be shown if RIPK1 cleavage was a direct cause for the dissociation of RIPK1 from complex I related to reduced Ser166 phosphorylation [24]. By contrast, the slight increase of phospho-Ser166 RIPK1 levels in *Bcl-3*^Hep^ mice following d-GalN/LPS challenge points to autophosphorylation of RIPK1 upon TNF-R1 stimulation in complex I, which is not by itself sufficient to induce cell death [24]. Although further research is required to elucidate the involvement of RIPK1, increased sensitivity of hepatocytes lacking RIPK1 to TNF-mediated apoptosis has been reported in several models [52, 53].

In summary, our results suggest that Bcl-3 exerts robust hepatoprotective effects early during ALF. The complexity around NF-κB signaling makes therapeutic modulation of Bcl-3 in the context of ALF a difficult target. However, the current analysis expands the field and provides the rational to further study Bcl-3 both as a target of pharmacotherapy or as a predictive biomarker in ALF.

## Materials and methods

### Animals

Transgenic mice with hepatocyte-specific overexpression of *Bcl-3* (Alfp-cre:bcl-3, *Bcl-3*^Hep^ mice) were generated as previously described [14]. RNA-sequencing (RNA-seq) analysis revealed that under 981 genes differentially expressed (@ FDR *p* < 0.05) in liver between naïve 8-week old *Bcl-3*^Hep^ and WT mice no significant differential expression of *Tnfrsf1a* encoding TNF-R1 was detected. However, gene expression of *Tnfrsf1b* (logFC = 0.63, *p* < 0.05, hit: 893) and *Fas* (logFC = 0.63, *p* < 0.01, hit: 485), which encode TNF-R2 and FAS (CD95/APO-1), resp., was significantly increased in *Bcl-3*^Hep^ livers [14]. *Bcl-3* knockout mice (*Bcl-3* KO) were kindly provided by Dr. Hana Algül (Technical University Munich, Germany). All animals were bred at the animal facility of the University Medical Center Mainz according to the criteria outlined by the “Guide for the Care and Use of Laboratory Animals”. Studies were approved by the Landesuntersuchungsamt Rheinland-Pfalz (Koblenz, Germany, G-18-1-066).

### Models of acute liver injury

Acute liver injury was induced in *Bcl-3*^Hep^ mice and WT littermates aged 10–12 weeks and of mixed sex by intraperitoneal (i.p.) injection of d-GalN (0.75 mg/g bodyweight, from d-(+)-galactosamine hydrochloride G1639, Carl Roth, Karlsruhe, Germany) and LPS (2.5 μg/g bodyweight, from *Escherichia coli* Serotype 026:B6, L-8274, Sigma-Aldrich, Hamburg, Germany) according to published protocols (average weight of the mice: 22 ± 1 g) [54, 55]. Age-matched controls received phosphate-buffered saline (PBS) injections. If indicated, necrostatin (Nec-1) was injected i.p. at a dose of 2 mg/kg body weight 1 h before d-GalN/LPS challenge. Blood and liver tissue were harvested at 4 or 6 h after d-GalN/LPS administration and preserved for evaluation of liver injury using established standard operating procedures. In separate experiments, survival rates were monitored up to 8 h after i.p. injection of d-GalN/LPS. In a second model of acute liver injury, female *Bcl-3*^Hep^ and WT mice aged 10–12 weeks were treated with agonistic FAS (CD95/APO-1) receptor antibody Jo2 (0.15 μg/g bodyweight, BD Pharmingen, Heidelberg, Germany, Cat# 554254) using intravenous (i.v.) tail vein injection [30]. *Bcl-3*^Hep^ and WT mice were randomly divided into the experimental and control groups.

### Serological analysis

Serum was obtained by cardiac puncture from anesthetized mice and serum ALT and AST levels were measured using a standard clinical analyzer (Hitachi 917, Roche, Mannheim, Germany). Serum cytokine levels were measured by BD cytometric bead array (CBA) Mouse Flex Sets (BD Biosciences, Heidelberg, Germany) using a BD FACS Canto II flow cytometer (BD Biosciences). Analysis was performed by FCAP Array^TM^v3 analysis software (Soft Flow, St. Louis Park, MN, USA).

### Histological analyses and immunohistochemistry

For histological examination representative liver sections were cut, fixed in 4% paraformaldehyde–PBS, embedded in paraffin, and stained with H&E using standard protocols and evaluated blinded by an expert hepatopathologist (BKS). Immunohistochemistry for activated (cleaved) caspase 3 (antibody from Cell Signaling Technology Inc., Danvers, MA, USA, Cat# 9664) was performed and analyzed as previously described [17, 30]. Immunocytochemistry using an NF-κB p65 antibody (Cell Signaling, Cat# 4764) was performed according to published protocols [3]. Representative pictures were obtained using an Olympus BX45 microscope (Olympus Deutschland, Hamburg, Germany) with a Jenoptik PROGRES GRYPHAX camera (Micro Optimal, Meerbusch, Germany) and the Olympus Image Analysis Software analySIS docu (Olympus Deutschland).

### Immunoblotting

Proteins were isolated and separated as previously described [56]. Primary antibodies included: BAK, BAX, BCL-X_L_, total and phospho-STAT3 (all Santa Cruz Biotechnology, Santa Cruz, CA, USA, Cat# sc-7873, sc-7480, sc-8392, sc-483, sc-8059), BID, c-FLIP, total and cleaved caspase 8, COX IV, cytochrome c, IKK-β, NF-κB p65, phospho-NF-κB p65 (Ser536), p44/42 MAPK (ERK1/2), phospho-p44/42 MAPK (ERK1/2) (Thr202/Tyr204), total and phospho-RIPK1 (Ser166), SAPK/JNK, phospho-SAPK/JNK (Thr183/Tyr185), XIAP (all Cell Signaling Technology Inc., Cat #2003, 3210, 4927, 9429, 4844, 4272, 2370, 4764, 3033, 9102, 9101, 3493, 53286, 9252, 4668, 2042), GAPDH (EnoGene, New York, NY, USA, Cat #E1C603) and tubulin (Abcam, Cambridge, MA, USA, Cat #ab4074). Membranes were exposed to anti-mouse (DAKO Denmark A/S, Glostrup, Denmark, Cat #P0447) or anti-rabbit (Santa Cruz Biotechnology, Cat #sc-2054) secondary antibodies conjugated with horseradish peroxidase. PaperPort Professional software v14.0 (Nuance Communications Germany, München, Germany) was used for image acquisition and the Adobe Acrobat Professional software program (Adobe Systems Incorporated, San Jose, CA, USA) was used to cut immunoblot images to size. No post-processing of images was performed. Densitometric analysis was performed using National Institutes of Health ImageJ software. Original, uncropped blots are presented in Supplementary Figure 5.

### Determination of caspase activity

Caspase 3, 8 and 9 activity was determined in whole liver tissue in duplicate experiments (Lysis buffer: 20 mM Tris/HCl pH 8.0, 5 mM EDTA, 0.5 % Triton X, cOmplete Mini protease inhibitor cocktail (Roche, Indianapolis, IN, USA)). 50 µl of tissue lysate (4 mg/ml) were used with an assay mixture containing 50 µl 2x reaction buffer (50 mM HEPES pH 7.5, 100 mM NaCl, 20% glycerol, 0.1% 3-[(cholamidopropyl-)dimethylammonio]-1-propanesulfonate (CHAPS), 10 mM DTT) and 5 µl 4 mM chromogenic peptide substrate (Ac-DEVD-AFC, Ac-IETD-AFC, or Ac-LEHD-AFC for caspase 3, 8, and 9, respectively, all Biomol, Hamburg, Germany) and incubated at 37 °C for 2 h without light. Cleavage was monitored colorimetrically at 405 nm.

### Determination of the malondialdehyde (MDA) content

MDA levels in whole liver tissue were detected and quantitated using the Lipid Peroxidation (MDA) Colorimetric Assay Kit (BioVision, Milpitas, CA, USA) according to the manufacturer’s instructions.

### Determination of the NF-κB activity

Activity of the NF-κB subunits p50, p52, and p65 was measured in duplicates using the TransAM NF-κB Family Kit (Active Motif, Carlsbad, CA, USA).

### Quantitative real-time (qRT-) PCR

Isolation of total RNA from snap frozen liver tissue, cDNA synthesis and qRT-PCR were performed as previously described [30]. All samples were performed in duplicates. Roche LightCycler software (LightCycler 480 Software Release 1.5.0) was used to perform advanced analysis relative quantification using the 2^(−ΔΔC(T))^ method. Expression data were normalized to the housekeeping gene *Gapdh* (Qiagen, Hilden, Germany) and the mean of PBS-treated WT mice was considered 1. Primer sequences (all Eurofins Genomics, Ebersberg, Germany) are detailed in Supplementary Table 2.

### Isolation of primary hepatocytes and ex vivo stimulation

Hepatocytes were isolated and cultured as previously described [56]. After 24 h, cells were treated with the gene transcription inhibitor actinomycin D (ActD, 200 ng/mL) in combination with murine TNF (10 ng/mL, both from Sigma-Aldrich, Hamburg, Germany) or Jo2 (50 ng/mL, BD Pharmingen), to induce death receptor-mediated apoptosis. Where indicated the pan caspase inhibitor zVAD (50 µM), the JNK inhibitor SP600125 (100 µM, both from Enzo Life Sciences, Lörrach, Germany), or the IKK inhibitor BAY-11-7082 (10 µM, Calbiochem, EMD Chemicals, Inc., San Diego, CA, USA) was added 1 h prior to ActD/TNF or Jo2 treatment. Cell survival was assessed by MTT assay (Sigma-Aldrich) at the indicated time points.

### Statistical analysis

All statistical analyses were performed using GraphPad Prism 7 software (GraphPad Software, La Jolla, CA, USA). All results were initially submitted to Shapiro–Wilk normality test for normality and to Levene’s test for homogeneity of variance. Comparisons between experimental groups were carried out using the unpaired, two-tailed Student’s *t* test or the Mann–Whitney *U* test to determine statistical significance of differences. The significance-level α was adjusted using Holm’s sequential Bonferroni adjustment in analyses involving multiple comparisons. Results with a *p* value of < 0.05 were considered to be significant. All data are shown as mean ± standard error of mean (SEM) to determine the precision and differences of means and statistically significant values were assumed with *^/$^*p* < 0.05, **^/$$^*p* < 0.01, ***^/$$$^*p* < 0.001. Survival times were analyzed by Kaplan–Meier curves with *p* values assessed with log-rank (Mantel–Cox) test. Median survival was also calculated.

## Supplementary information

Graphical Abstract

Supplemental Material

AJ Checklist

## Data Availability

All data will be shared upon request.
