# Supplementary material for: Hepatocyte Bcl-3 protects from death-receptor mediated apoptosis and subsequent acute liver failure
Source: Cell Death Dis. 2022 May 31;13(5):510. doi: 10.1038/s41419-022-04946-y (PMC9156769; doi:10.1038/s41419-022-04946-y)
Supplement: Supplementary file 1 — Graphical Abstract [file 41419_2022_4946_MOESM1_ESM.pptx]

## Slide 1
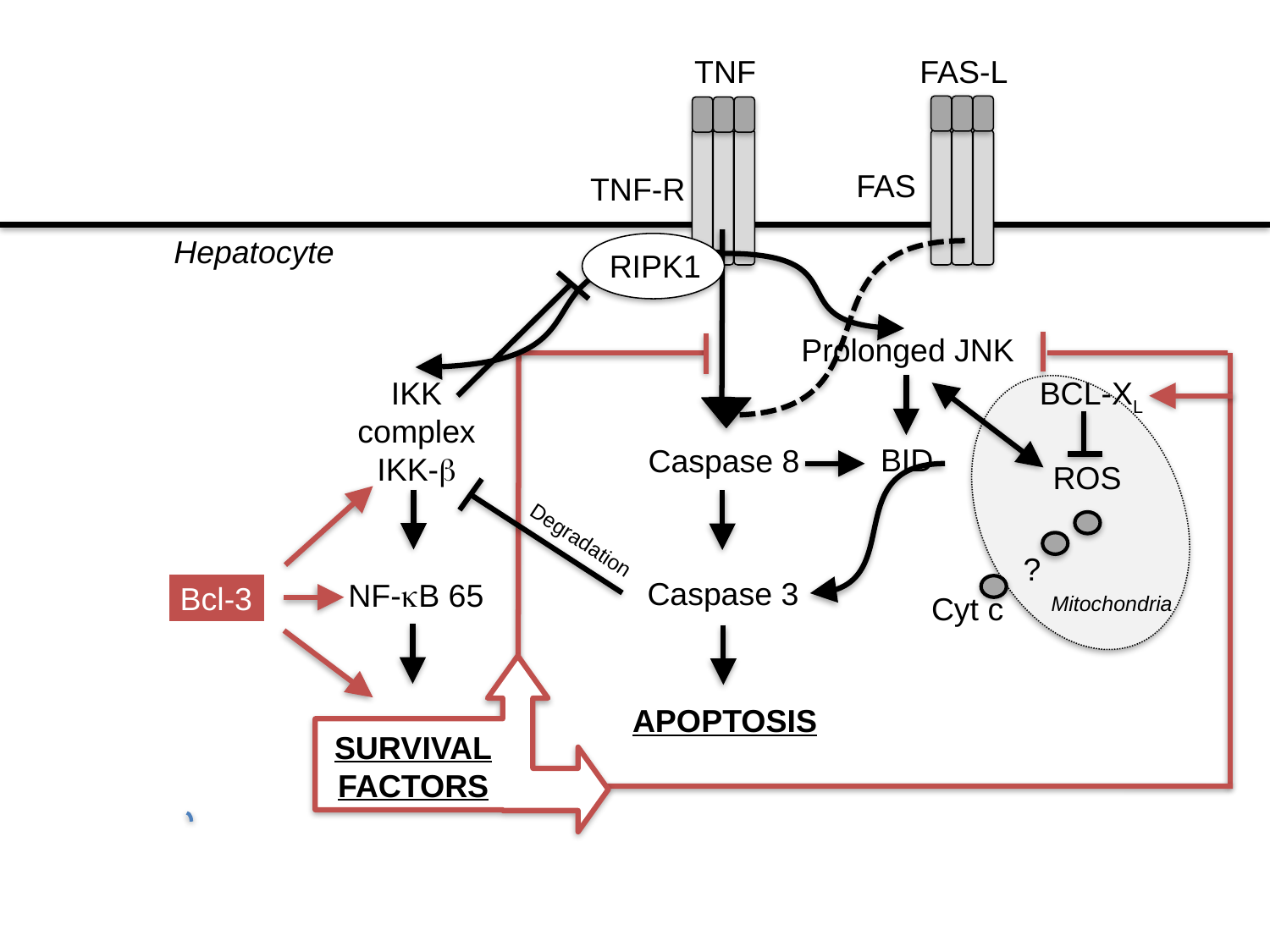

TNF
FAS-L
FAS
TNF-R
Hepatocyte
RIPK1
Prolonged JNK
IKK complex
IKK-b
BCL-XL
BID
Caspase 8
ROS
Degradation
?
Caspase 3
NF-kB 65
Bcl-3
Cyt c
Mitochondria
APOPTOSIS
SURVIVAL
FACTORS
