# Supplementary material for: Hepatocyte Bcl-3 protects from death-receptor mediated apoptosis and subsequent acute liver failure
Source: Cell Death Dis. 2022 May 31;13(5):510. doi: 10.1038/s41419-022-04946-y (PMC9156769; doi:10.1038/s41419-022-04946-y)
Supplement: Supplementary file 2 — Supplemental Material [file 41419_2022_4946_MOESM2_ESM.docx]

**Supplemental Material**

**Supplementary figure 1: D-GalN/LPS challenge induced systemic and hepatic inflammation equivalent in *Bcl-3*^Hep^ and WT mice.** *Bcl-3*^Hep^ and WT mice were i.p. injected with D-GalN/LPS or PBS and (A) serum concentration and (C) relative hepatic mRNA expression of inflammatory cytokines were assessed after 4 h. (B) Time course analysis of TNF secretion in D-GalN/LPS-challenged mice. (D) Expression levels of phosphorylated and total STAT3 were determined in whole liver tissue lysates at 4 h post D-GalN/LPS by immunoblotting. Data in A represent means of n=7 WT + D-GalN/LPS, n=9 *Bcl-3*^Hep^ + D-GalN/LPS and n=6 PBS-treated controls per genotype ± SEM. Data in B represent means of n=6 mice per genotype ± SEM at 1 h and 2 h post D-GalN/LPS and n=7 WT + D-GalN/LPS, n=9 *Bcl-3*^Hep^ + D-GalN/LPS ± SEM at 4 h post D-GalN/LPS. Data in C represent mean of n=6 mice/group ± SEM. ^$^ *p<.05*, ^$$^ *p<.01* for PBS vs. D-GalN/LPS according to Mann-Whitney *U* test (A-C). In D representative western blots with densitometric analysis are shown. There was no statistical difference between WT + D-GalN/LPS and *Bcl-3*^Hep^ + D-GalN/LPS in respect to the ratio phospho-STAT3/STAT3 according to unpaired, two-tailed Student’s *t*-test.


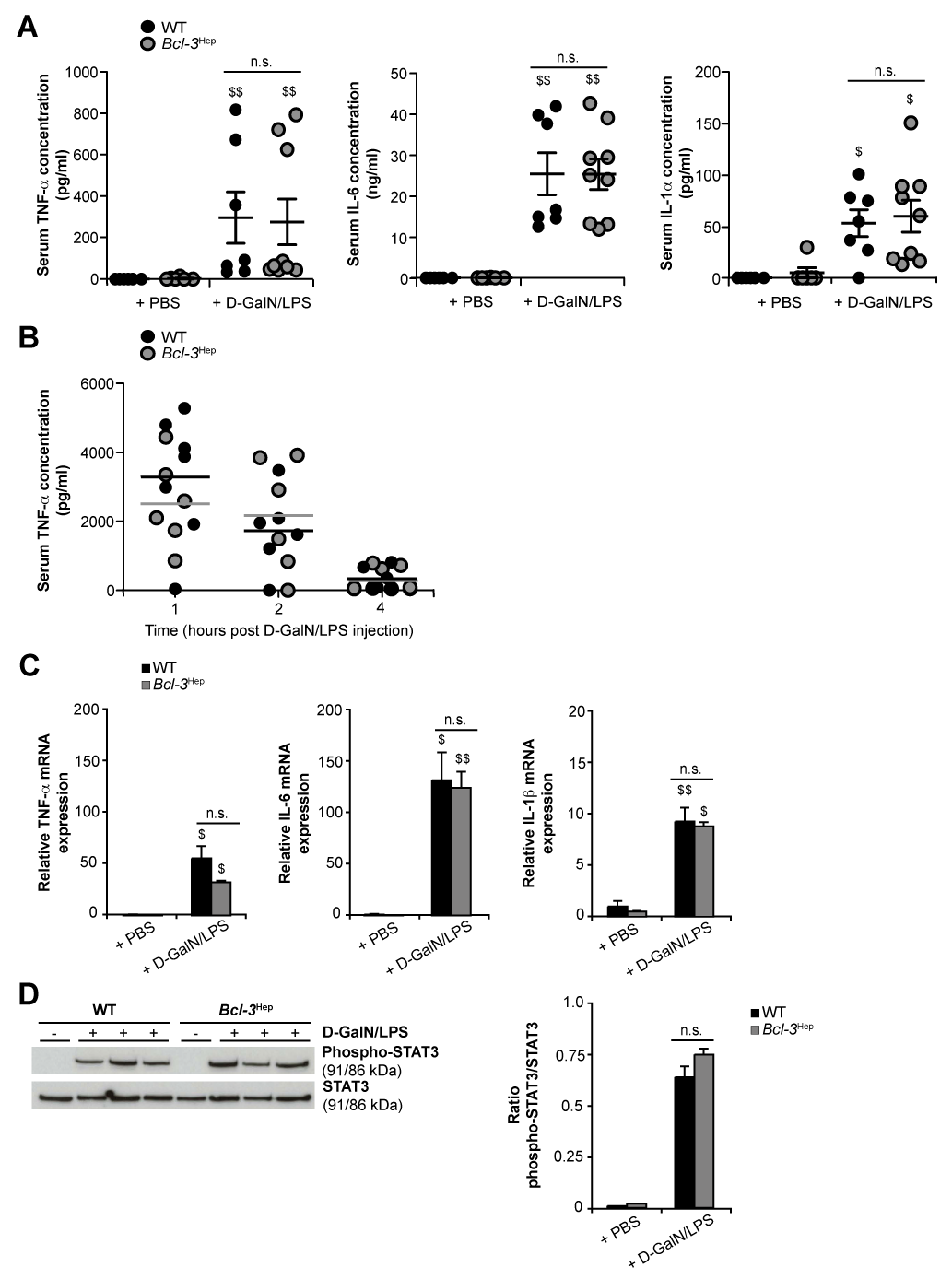


**Supplementary figure 2: Hepatic expression levels of the anti-apoptotic proteins BAX, BAK, c-FLIP_L/S_ and XIAP did not correlate with the reduced susceptibility of *Bcl-3*^Hep^ mice to D-GalN/LPS-induced hepatocellular injury.** Western blot analysis of (A) BAX, (B) BAK, (C) c-FLIP_L/S_, and (D) XIAP protein in whole liver tissue lysates from *Bcl-3*^Hep^ and WT mice at 4 h post D-GalN/LPS challenge and corresponding controls. GAPDH and tubulin served as protein loading controls. Representative western blots with densitometric analysis are shown.


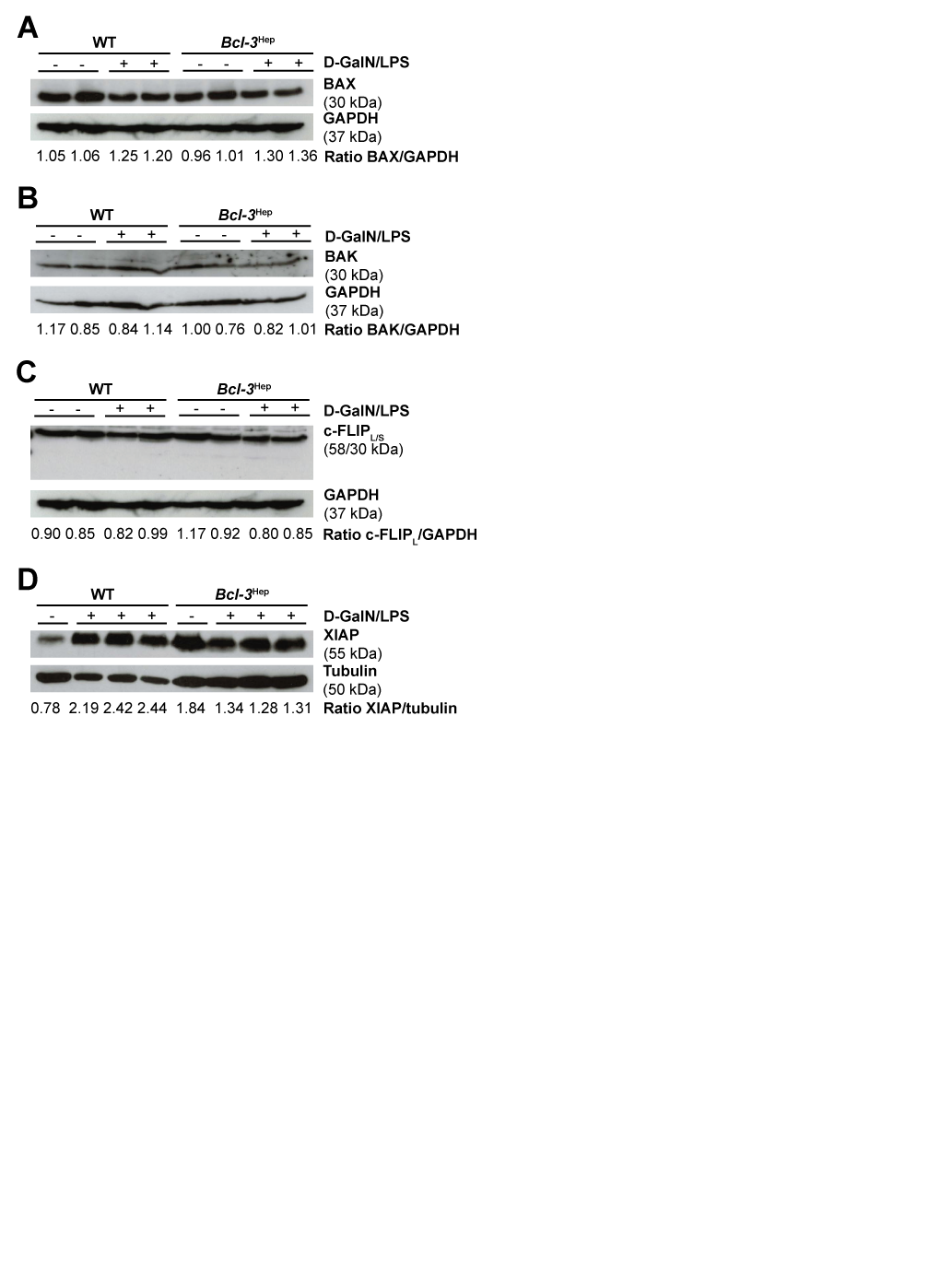


**Supplementary figure 3: Hepatic NF-**κ**B p65 expression visualized by confocal microscopy**. *Bcl-3*^Hep^ and WT mice were i.p. injected with D-GalN/LPS or saline and hepatic NF-κB p65 expression was visualized by confocal microscopy after 4 h (scale bar: 50 µm). Cell nucleus was stained with DAPI. Representative pictures are shown.


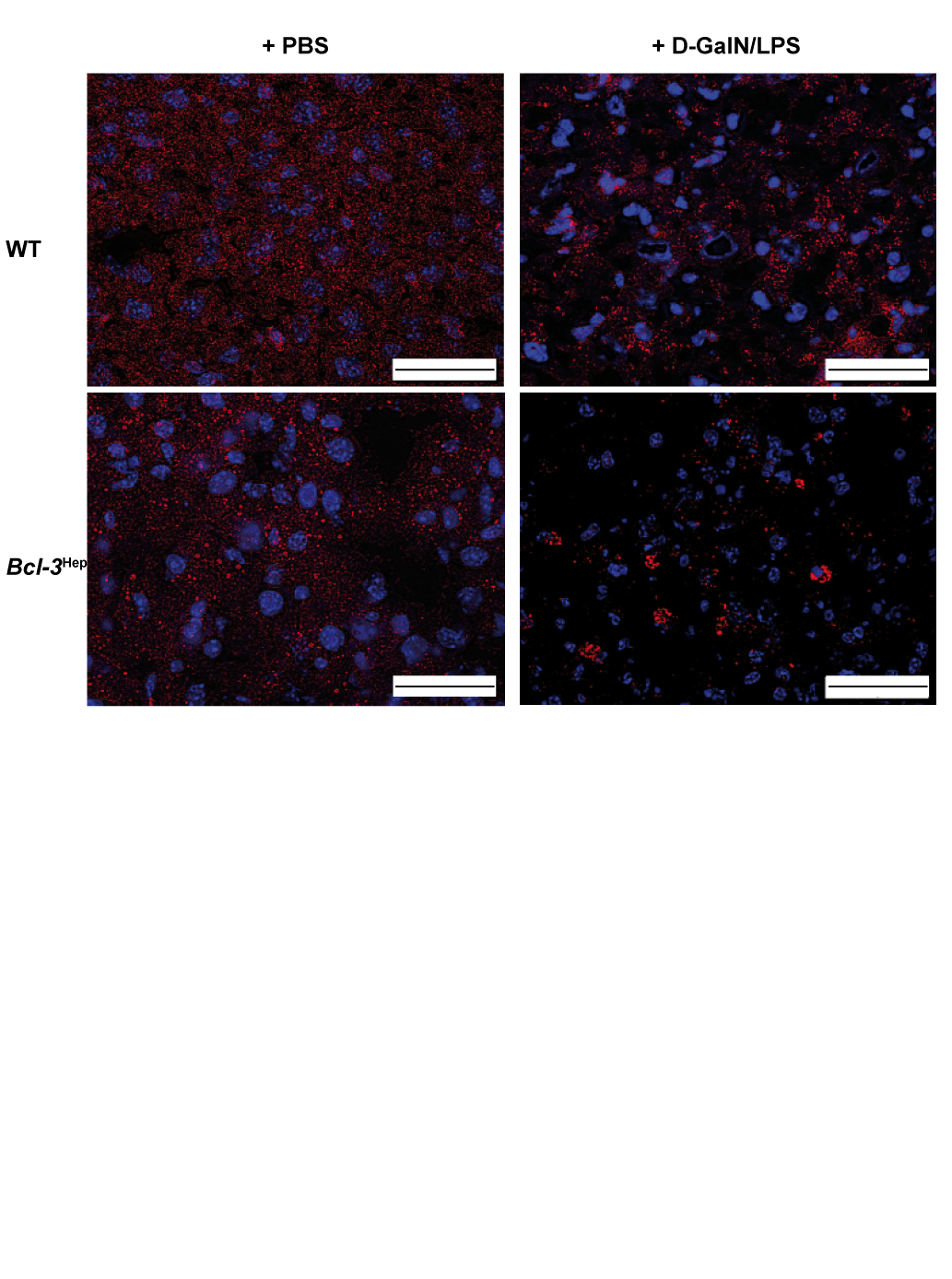


**Supplementary figure 4: Comparable JNK activation in hepatic tissue of Jo2-challenged *Bcl-3*^Hep^ and WT mice.** Hepatic JNK activity in *Bcl-3*^Hep^ and WT mice was analyzed by immunoblotting in whole liver tissue lysates at 4 h post Jo2 using specific antibodies against phospho-JNK (Thr183/Tyr185) and total JNK. Tubulin served as protein loading controls. A representative western blot with densitometric analysis is shown.


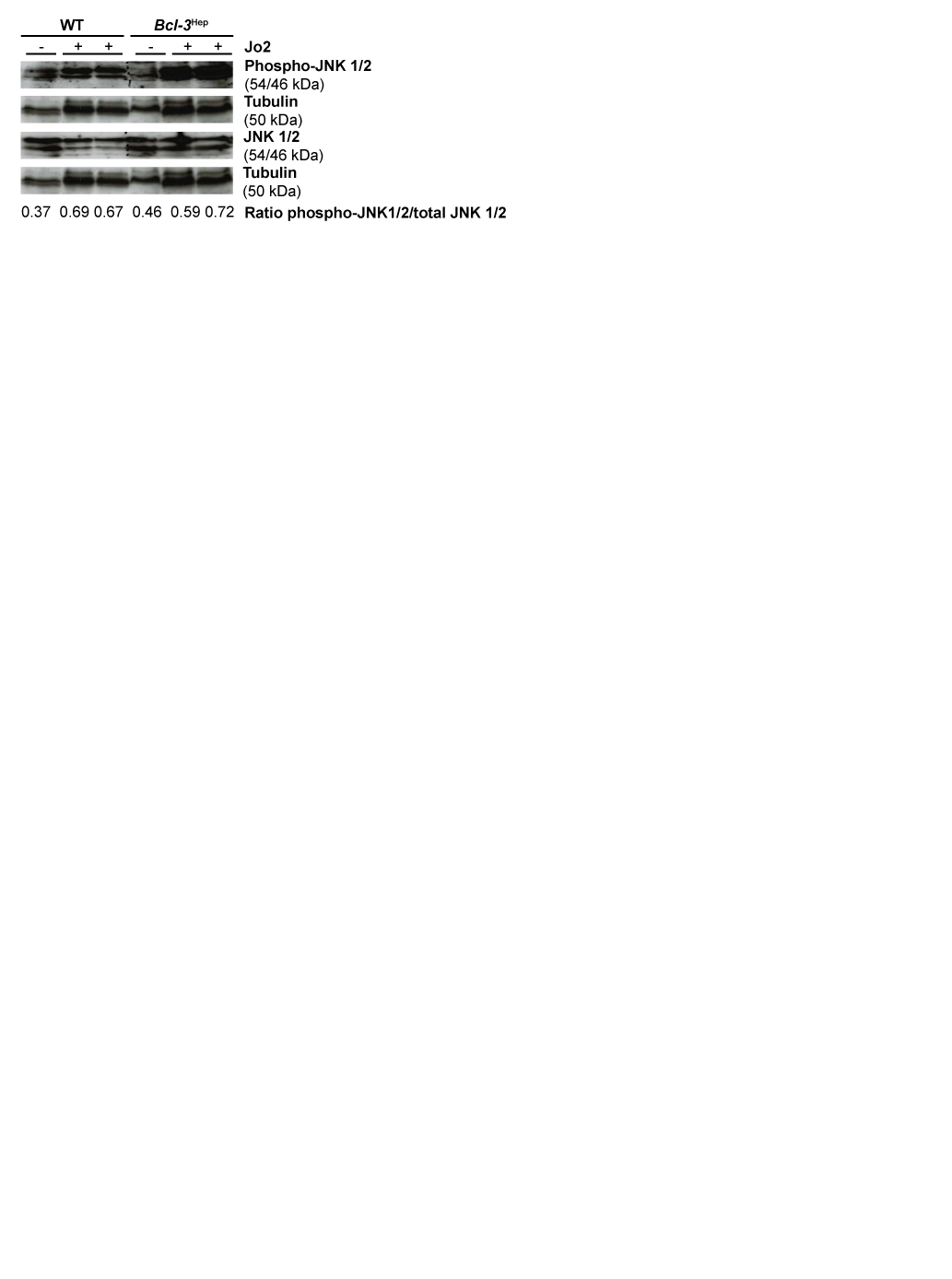


**Supplementary figure 5: Original, uncropped blots of (A) figure 2A, (B) 2D, (C) 2E, (D) 2F, (E) 3A, (F) 3C, (G) 3D, (H) 5A, (I) 5B, (J) 6C, and (K) 6D.** Asteriks mark unspecific bands.

**
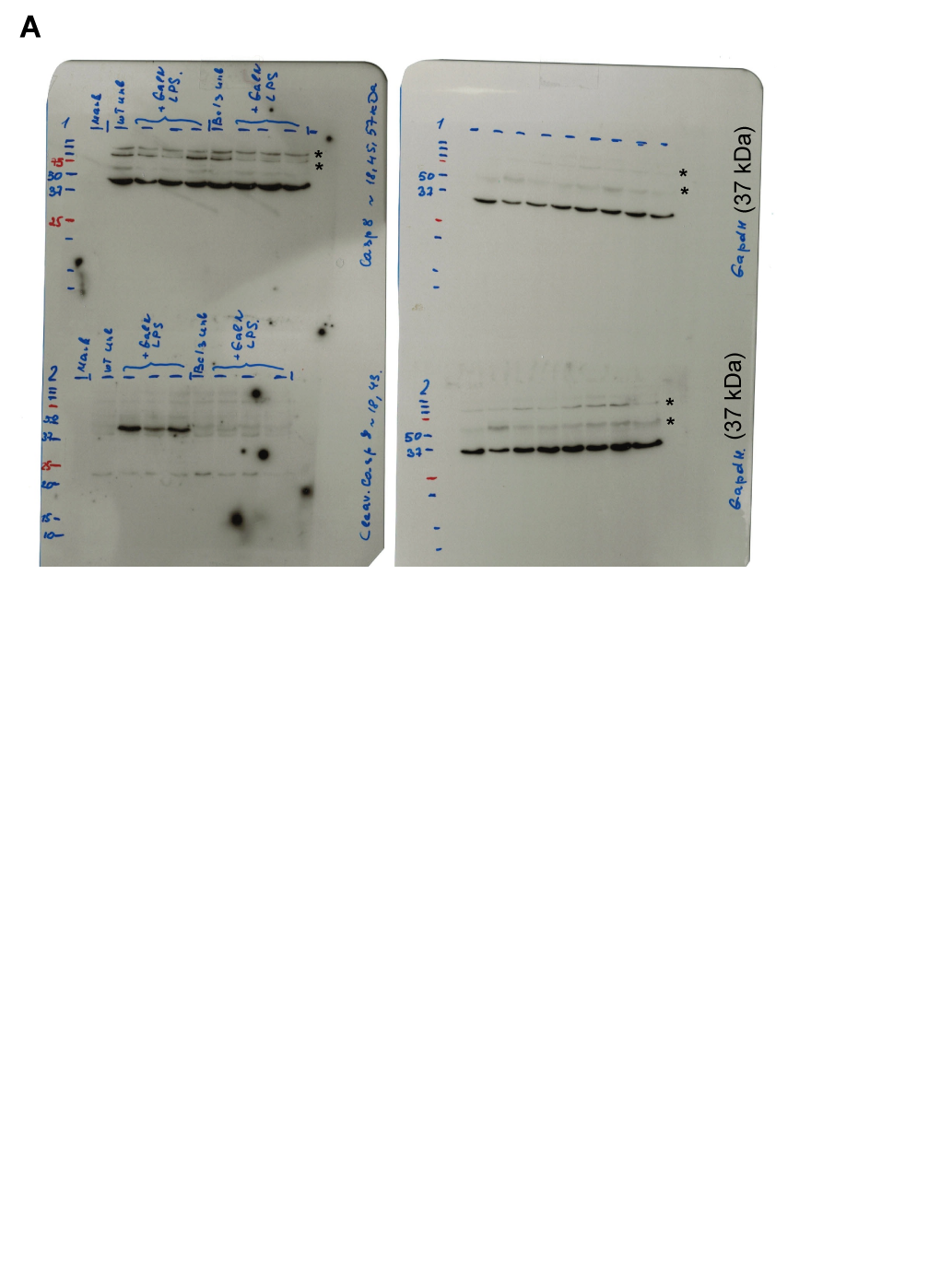
**

**
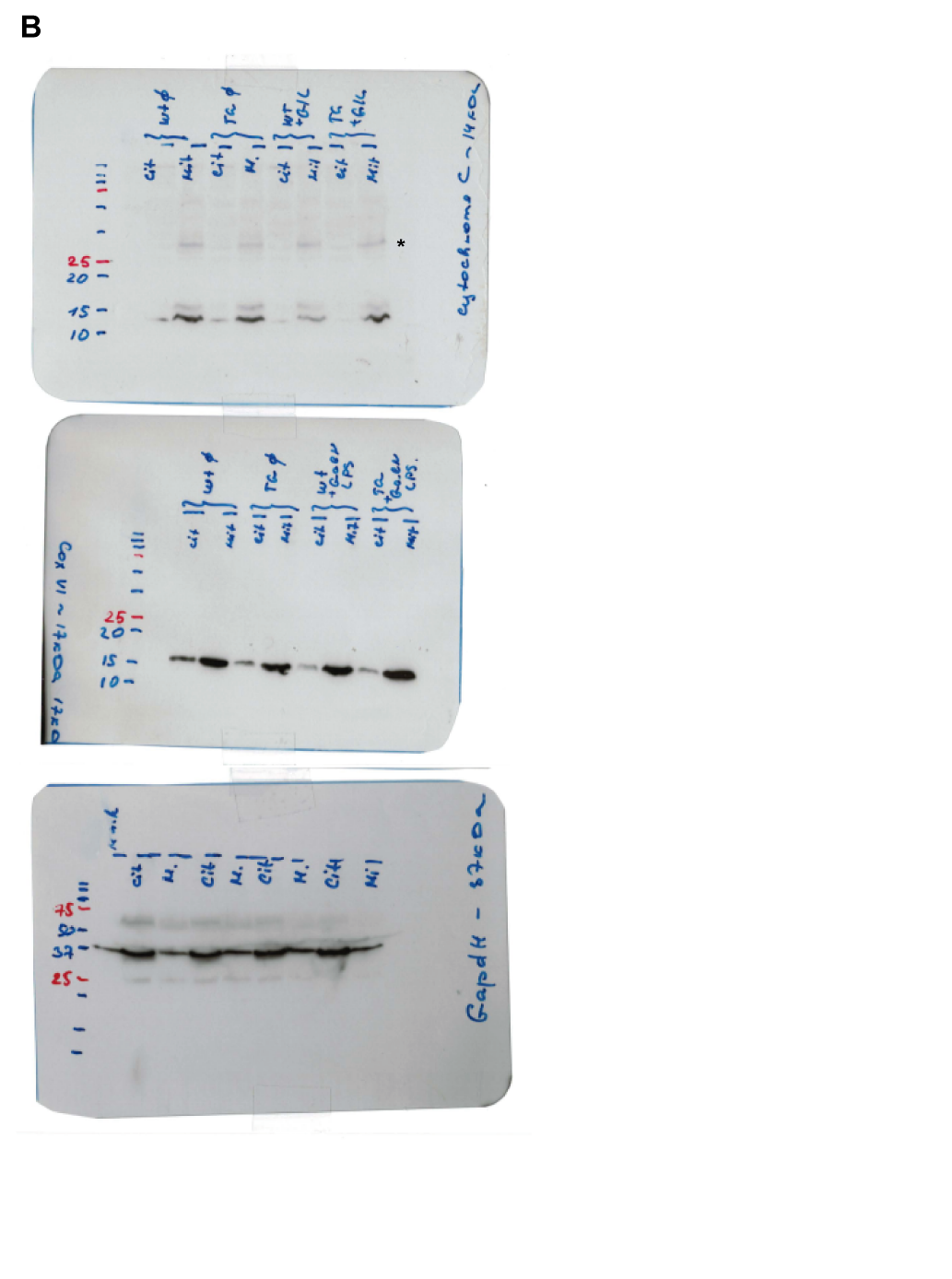
**

**
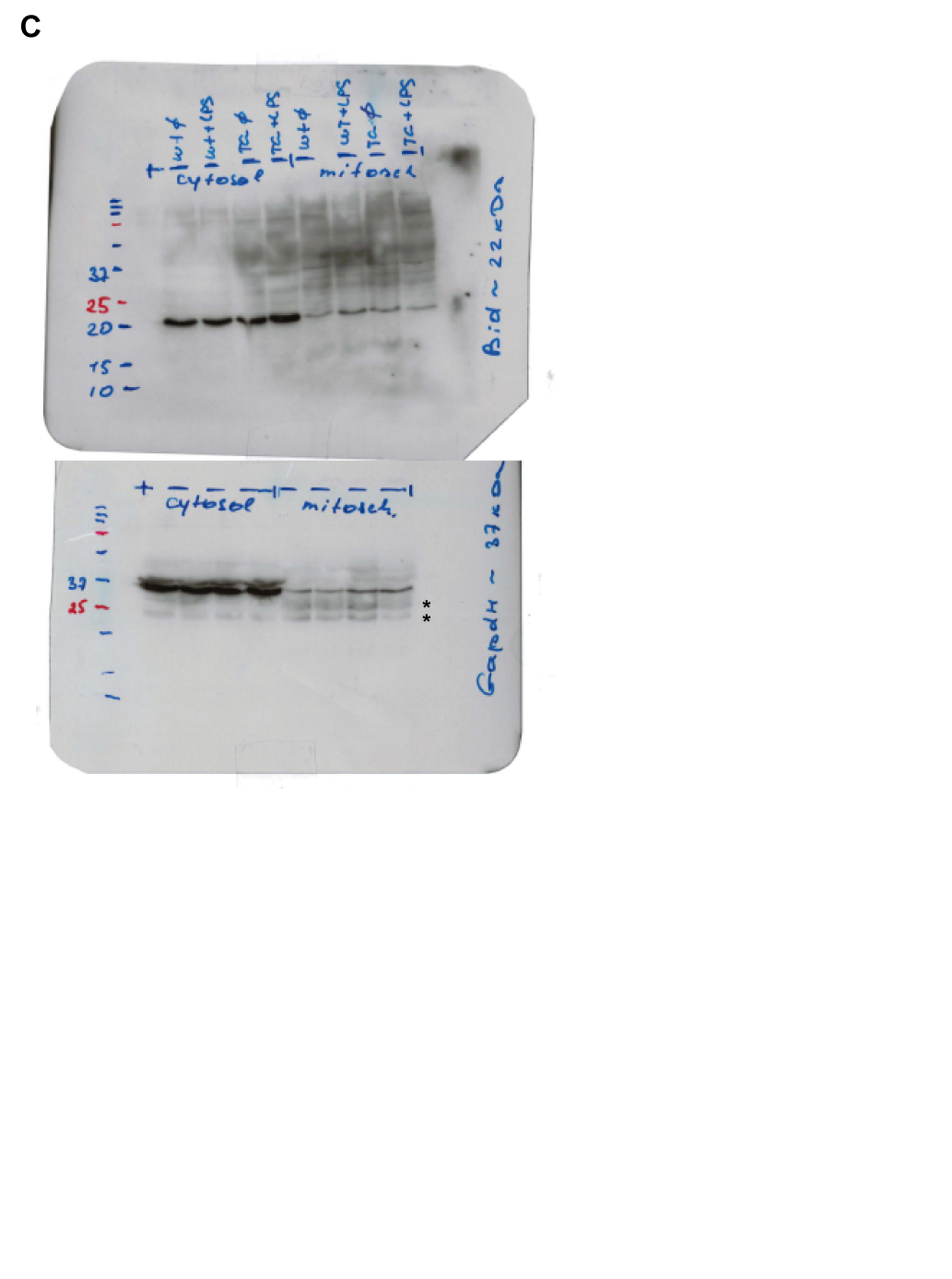
**

**
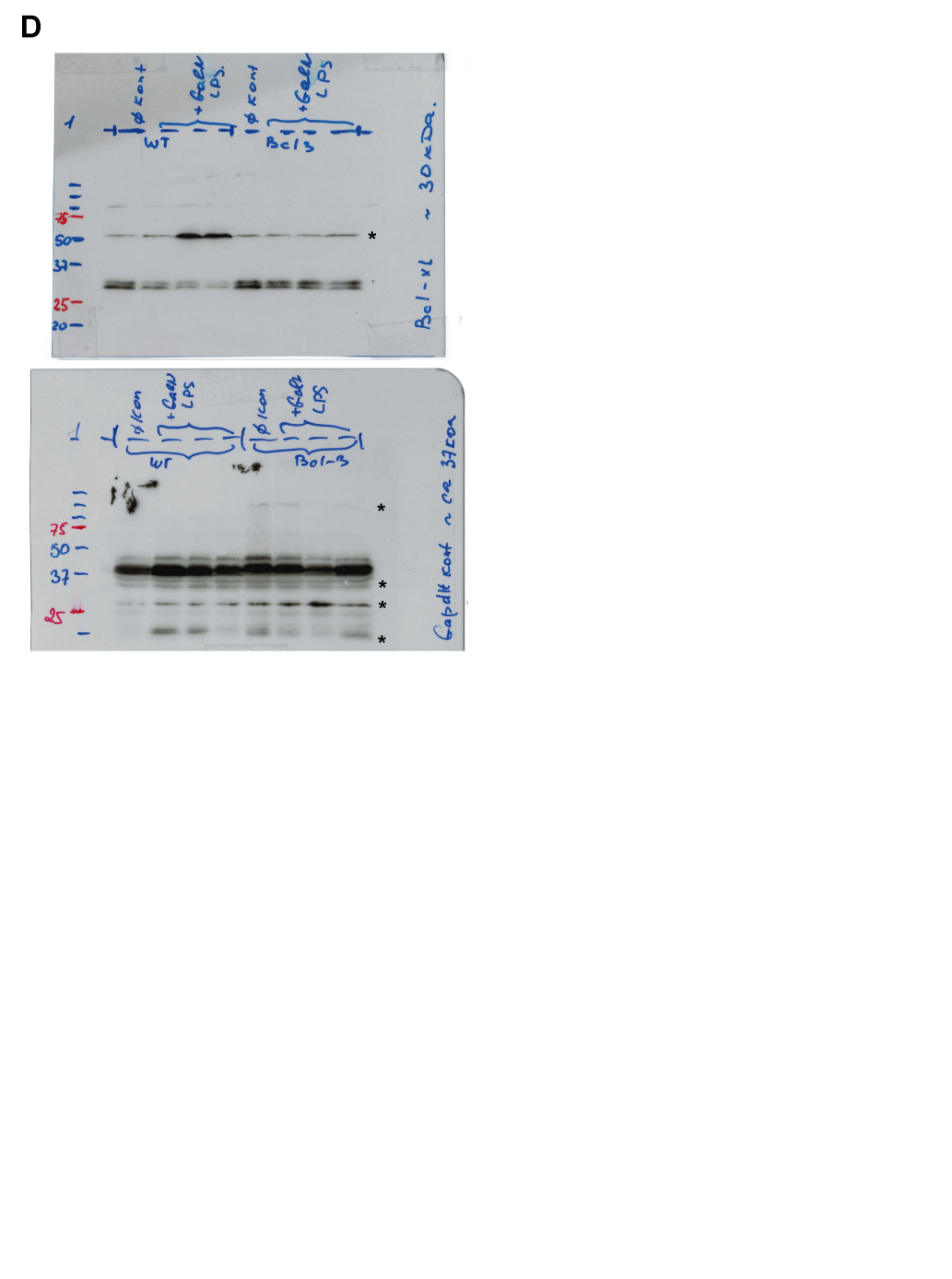
**

**
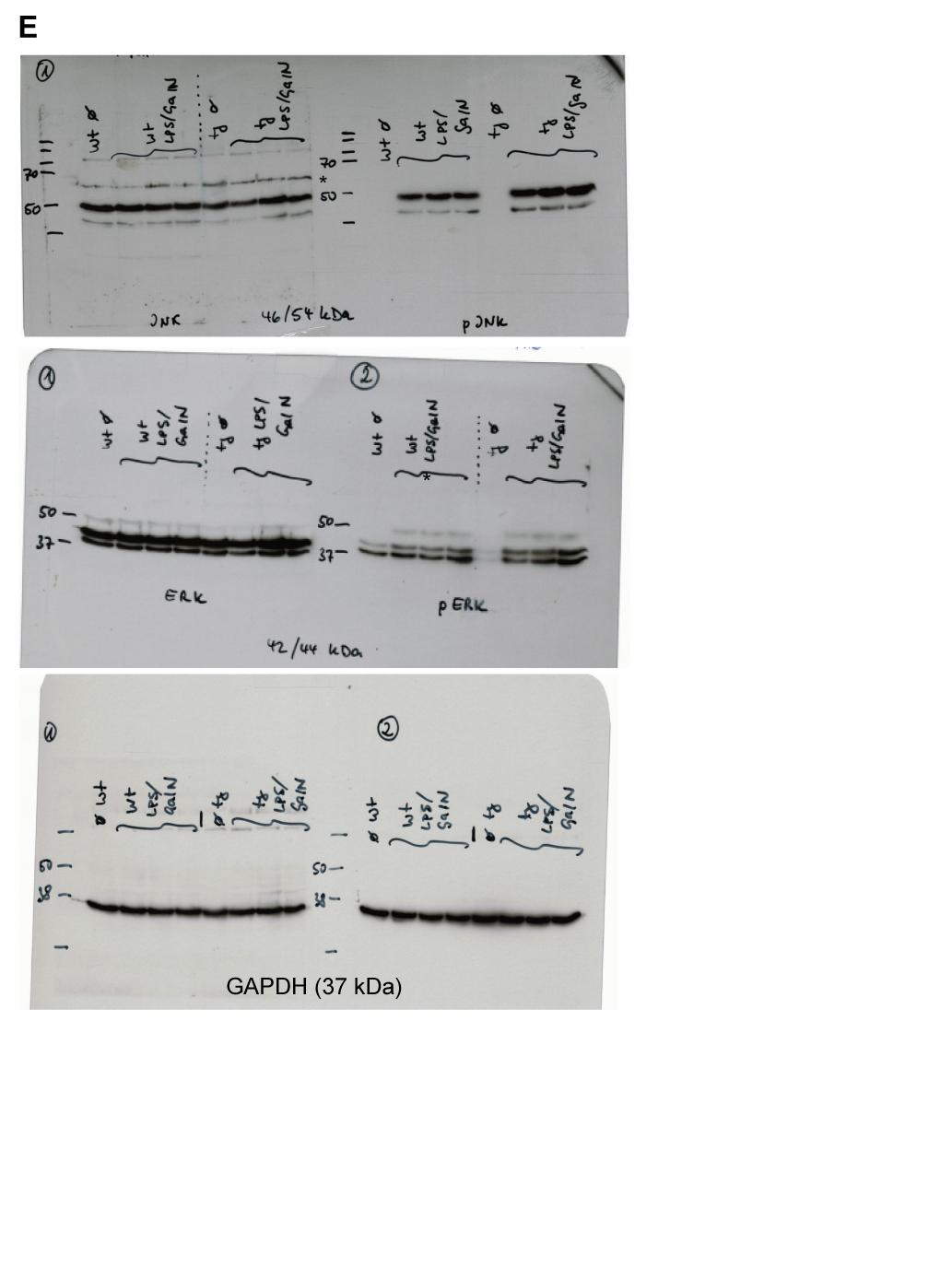
**

**
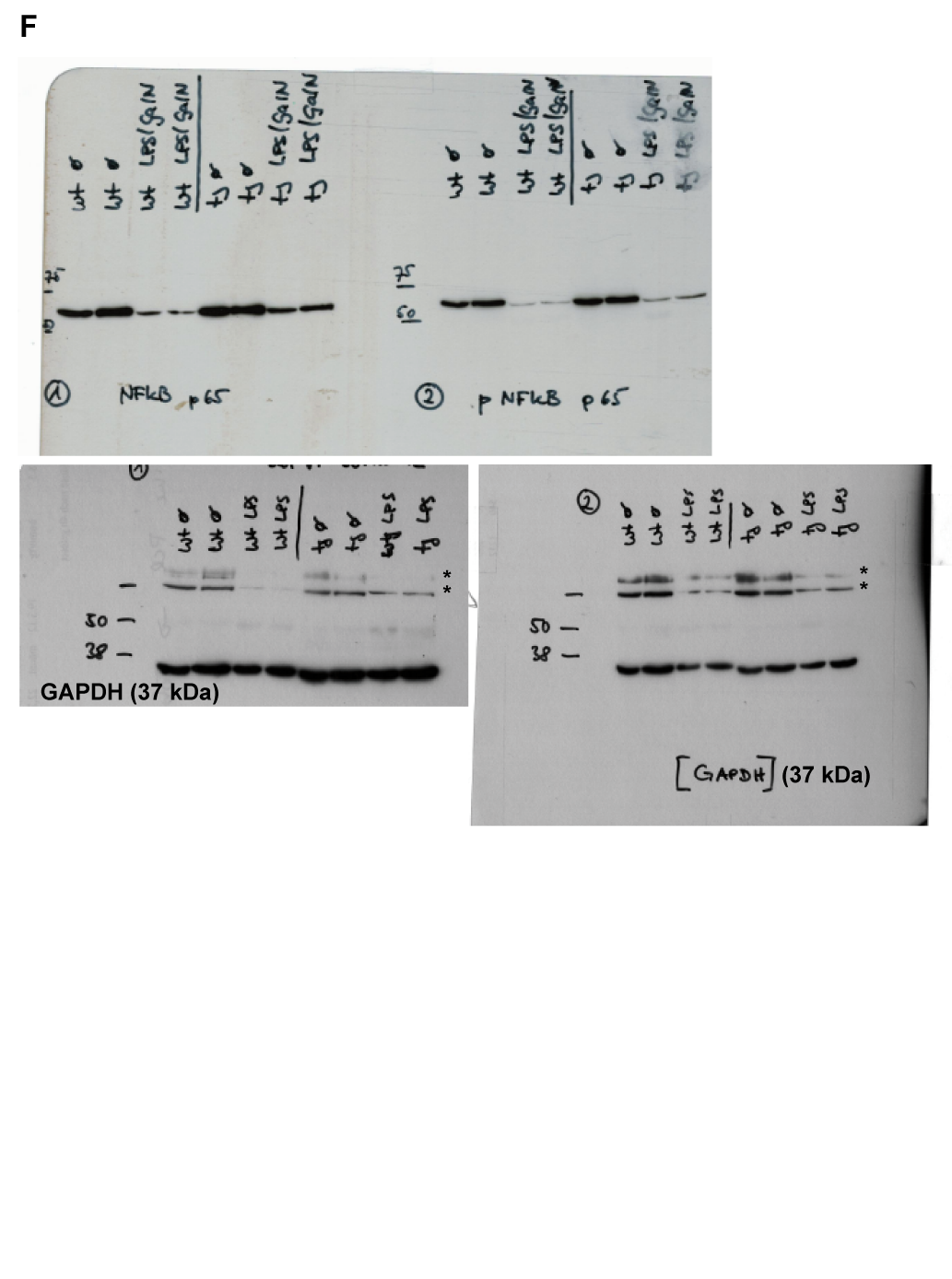
**

**
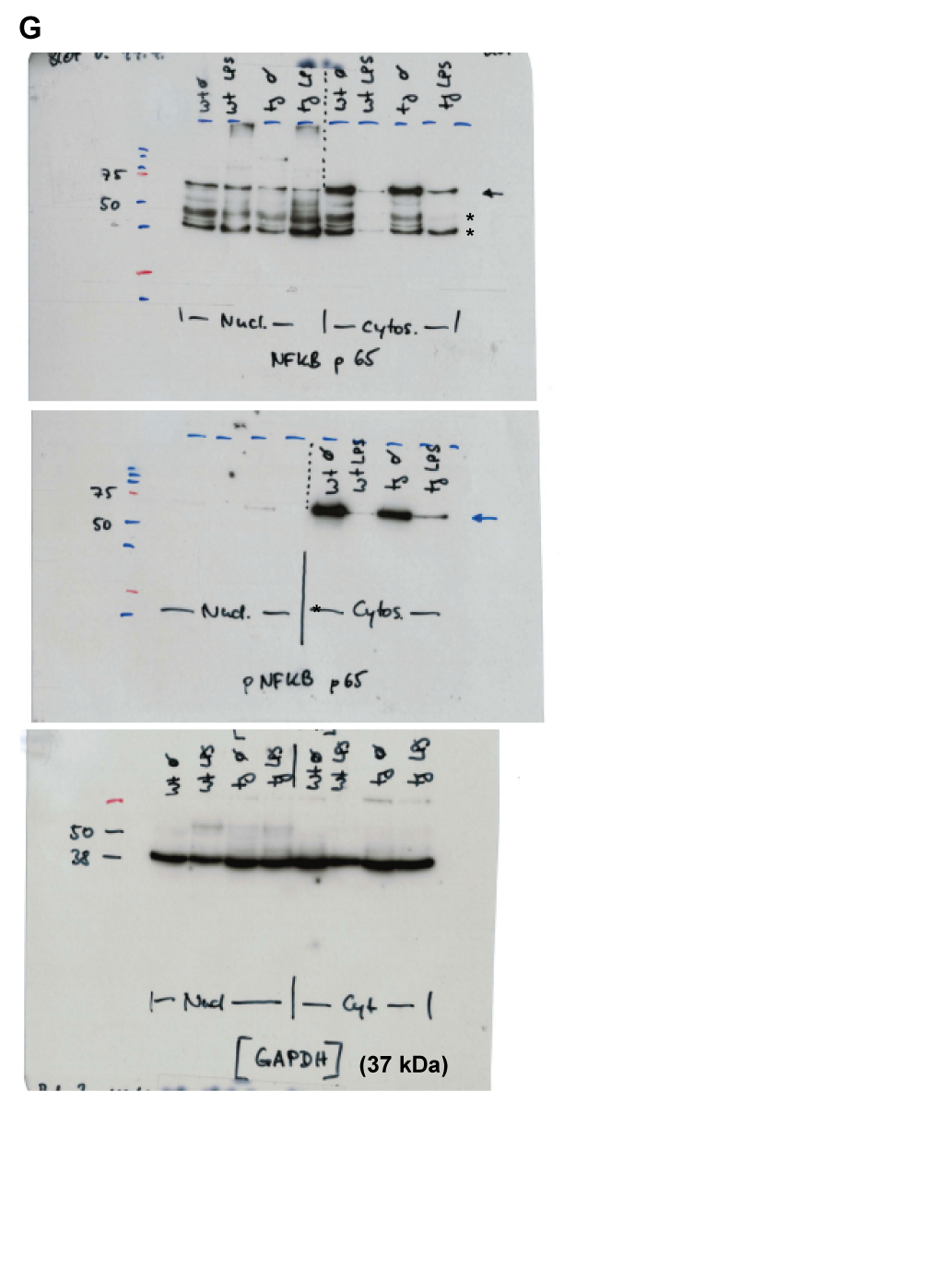

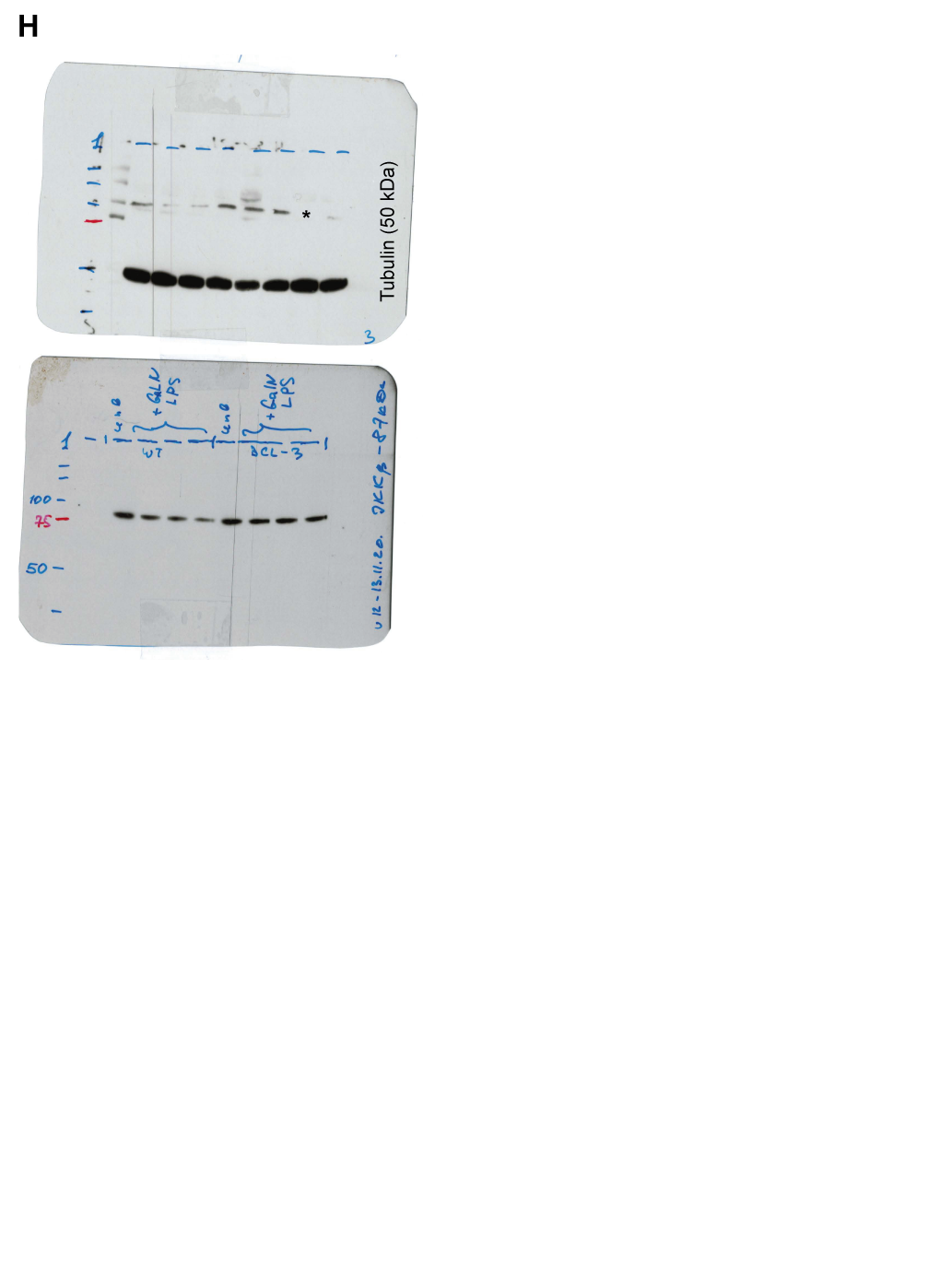
**

**
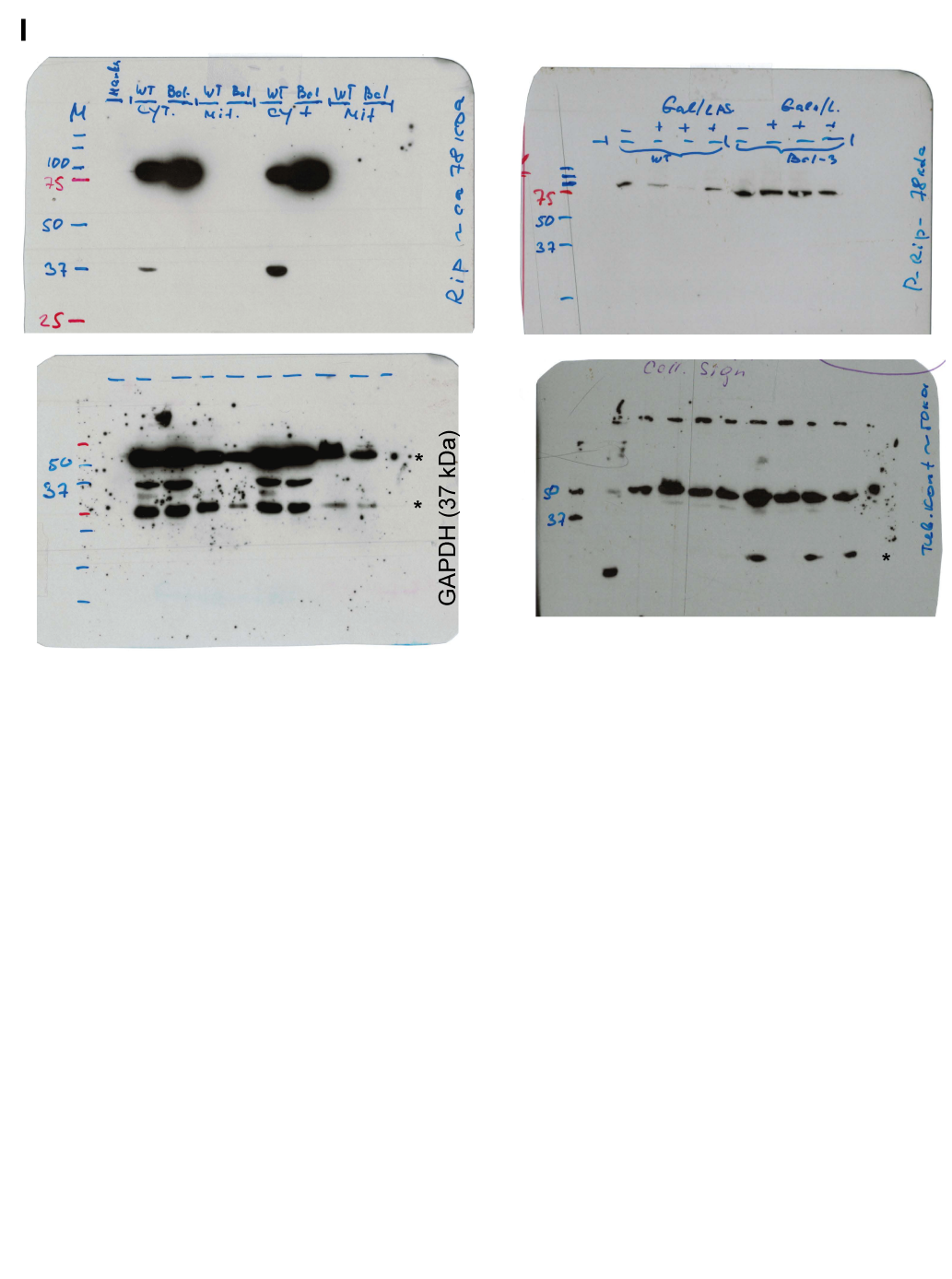
**

**
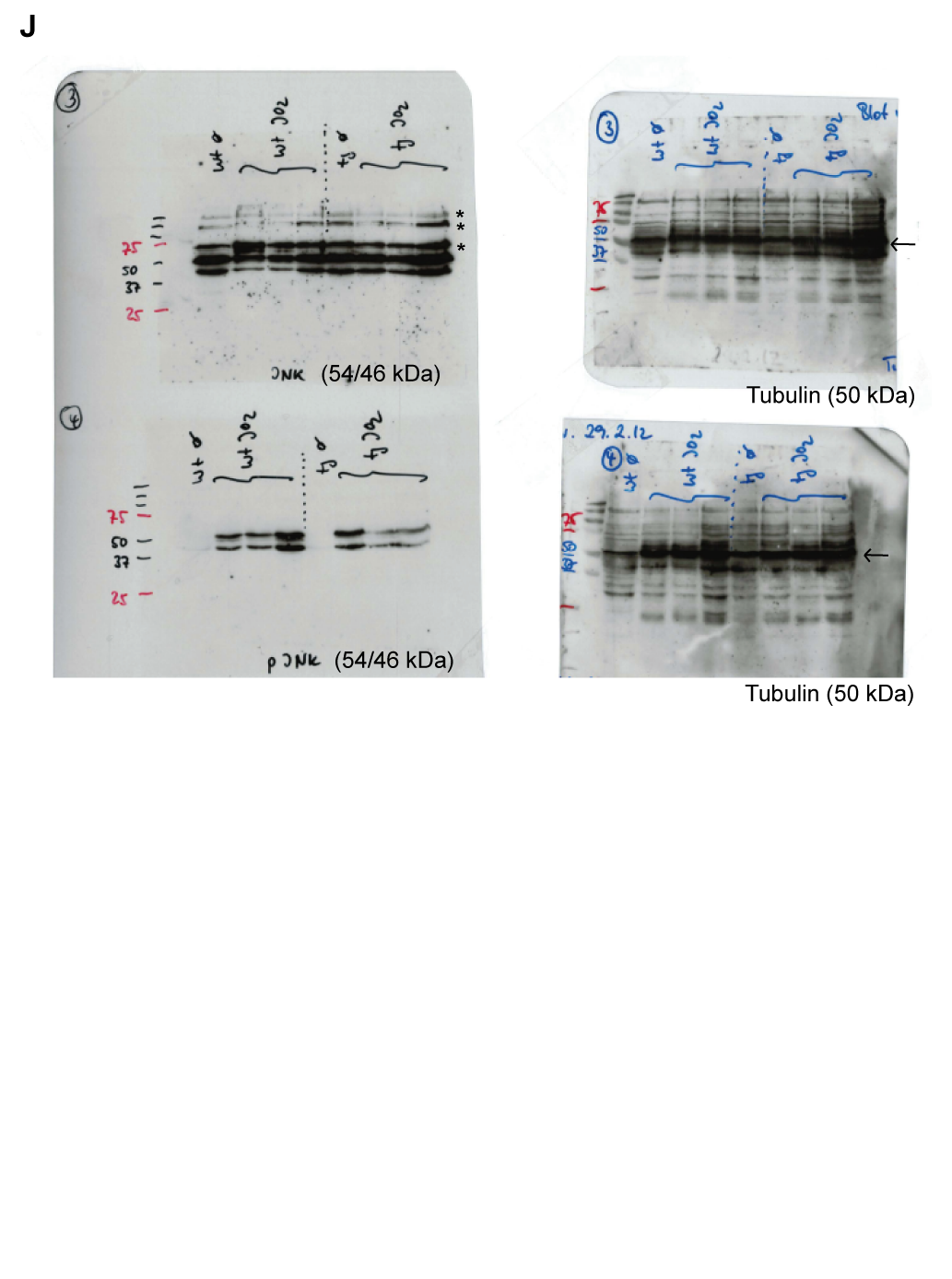
**

**
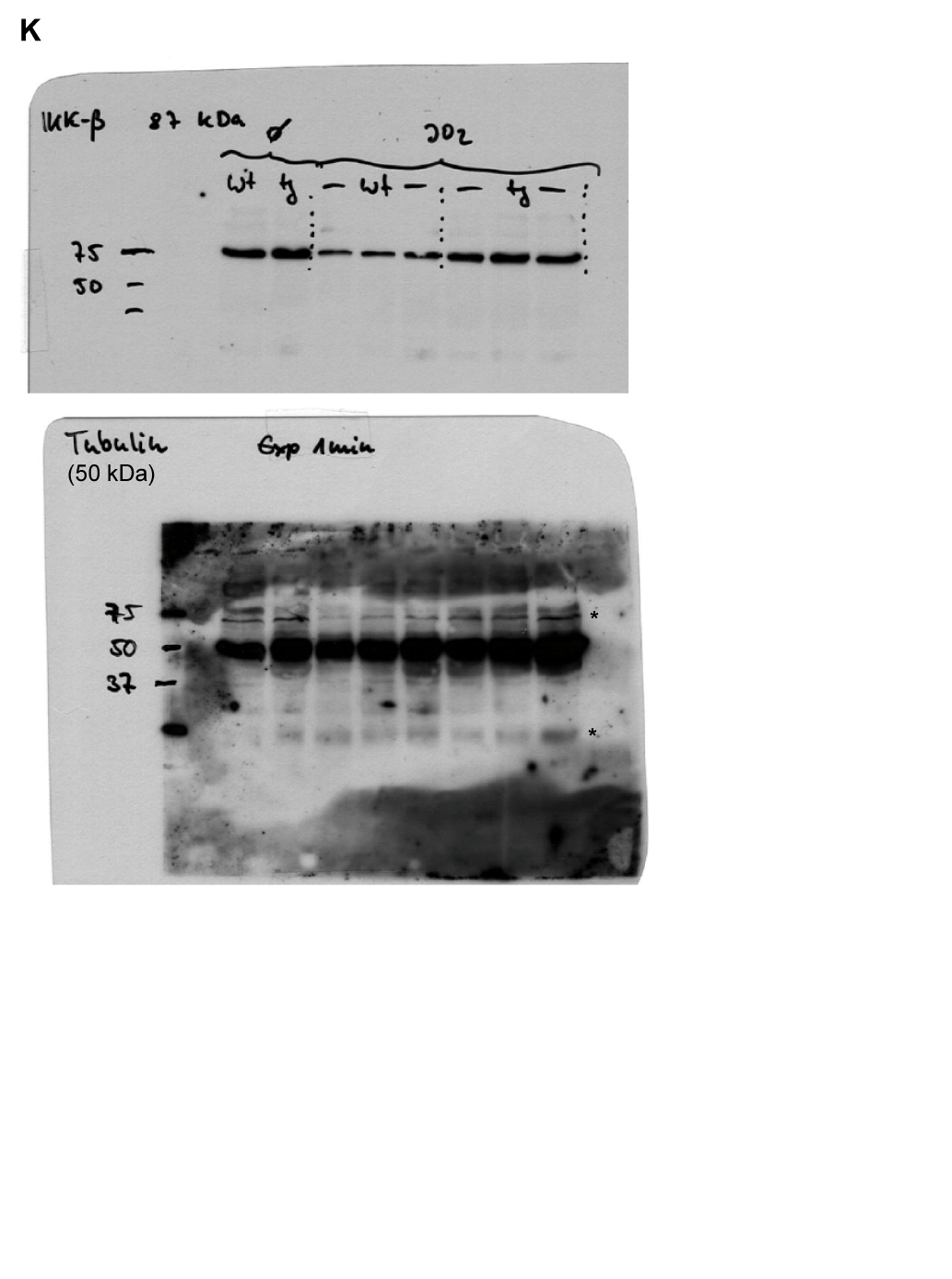
**

**Supplementary table 1: (A) Relative hepatic gene expression of RIPK1 and RIPK3 and (B) serum transaminase levels in *Bcl-3*^Hep^ and WT mice at 4 h post D-GalN/LPS challenge with and without necrostatin-1 (Nec-1) pretreatment and in control animals.** In A data represent means of n=6-9 mice/group ± SEM for RIPK1 and n=4-10 mice/group ± SEM for RIPK3. Data in B represent means of n=8 WT + Nec-1 + D-GalN/LPS and n=9 *Bcl-3*^Hep^ + Nec-1 + D-GalN/LPS ± SEM, whereby one WT mouse was already dead at this time point. *** *p<.001* for WT vs. *Bcl-3*^Hep^, ^$^ *p <.05*, ^$$^ *p <.01*, ^$$$^ *p<.001* for PBS vs. D-GalN/LPS or Nec-1 + D-GalN/LPS, and ^#^ *p <.05* , ^##^ *p <.01* for D-GalN/LPS vs. Nec-1 + D-GalN/LPS according to Mann-Whitney *U* test. There was no statistically significant difference between WT/*Bcl-3*^Hep^ + D-GalN/LPS vs. WT/*Bcl-3*^Hep^ + Nec-1 + D-GalN/LPS in respect of the parameters.

**(A)**

|  | **WT + PBS** | **WT + D-GalN/LPS** | **WT + Nec-1 + D-GalN/LPS** | ***Bcl-3*^Hep^ + PBS** | ***Bcl-3*^Hep^ + D-GalN/LPS** | ***Bcl-3*^Hep^ + Nec-1 + D-GalN/LPS** |
| --- | --- | --- | --- | --- | --- | --- |
| **RIPK1** | 1.00 ± 0.25 | 1.44 ± 0.19 | 2.76 ± 0.19 ^($$, ##)^ | 1.48 ± 0.06 | 1.36 ± 0.24 | 2.16 ± 0.20 ^($, #)^ |
| **RIPK3** | 1.00 ± 0.08 | 1.13 ± 0.14 | 2.28 ± 0.37 ^($, #)^ | 0.68 ± 0.08 | 0.94 ± 0.11 | 2.07 ± 0.44 ^($, #)^ |

**(B)**

|  | **WT + PBS** | **WT + D-GalN/LPS** | **WT + Nec-1 + D-GalN/LPS** | ***Bcl-3*^Hep^ + PBS** | ***Bcl-3*^Hep^ + D-GalN/LPS** | ***Bcl-3*^Hep^ + Nec-1 + D-GalN/LPS** |
| --- | --- | --- | --- | --- | --- | --- |
| **ALT (U/l)** | 20 ± 0 | 865 ± 125 ^($$$)^ | 2715 ± 1543 ^($)^ | 20 ± 0 | 331 ± 76 ^(^***^,^ ^$$$)^ | 1394 ± 838 ^($$)^ |
| **AST (U/l)** | 142 ± 33 | 1023 ± 137 ^($$$)^ | 1972 ± 929 ^($)^ | 156 ± 26 | 432 ± 70 ^(^***^,^ ^$)^ | 1337 ± 695 ^($)^ |

**Supplementary table 2: Forward and reverse primers used for qRT-PCR.**

| **Gene** | **Protein** | **Forward primer (5’-3’)** | **Reverse primer (5’-3’)** |
| --- | --- | --- | --- |
| *Bak1* | BAK | CAG CCC TGA ATT TTC GTA GAG AC | AGT GGA GAA AAA GGG TGG ATT GTA |
| *Bax* | BAX | AGG GTT TCA TCC AGG ATC GAG CAG | ATC TTC CAG ATG GTG AGC GAG |
| *Bcl2* | BCL-2 | GCC AGG GAA GAT GGC TGA GTC TG | TTG GAG CCG ACT CAA AGG CGG G |
| *Bcl2l1* | BCL-X_L_ | GGG GTC GCA TCG TGG CCT TT | AAG CGC TCC TGG CCT TTC CG |
| *Birc2* | cIAP1 | TCA CGC ACA GAA GAG CCA CGG | GCT GGG TGG CGC GAT ACC TT |
| *Cflar* | cFLIP_L_ | GCA GAA GCU CUC CCA GCA | UUU GUC CAU GAG UUC AAC GUG |
|  | cFLIP_S_ | UCC AGA AGU ACA CCC AGU CCA | CAC UGG CUC CAG ACU CAC C |
| *Il6* | IL-6 | AGT TGC CTT CTT GGG ACT GA | TTC TGC AAG TGC ATC ATC GT |
| *Mcl1* | MCL-1 | AAG AGG CTG GGA TGG GTT TGT | AGT CCC CTA TTG CAC TCA CAA G |
| *Nfkb1* | p50/p105 | TAG CGG CCG GAA GAG GGT CC | GAC ACC GTC TGT GCG TGG CA |
| *Rel* | c-Rel (Rel) | GGA GCG CGA AGA TTC GGG GG | TGC GCT CCC CTG GGA TGC TA |
| *Rela* | p65 (RelA) | CGC TTC TCT TCA ATC CGG T | GAG TCT CCA TGC AGC TAC GG |
| *Relb* | RelB | GTC TTT CCC CAC GAG GCT AT | ATC GAG CTT CGA GAC TGT GG |
| *Ripk1* | RIPK1 | GGA AGG ATA ATC GTG GAG GC | AAG GAA GCC ACA CCA AGA TC |
| *Ripk3* | RIPK3 | GGT AAA GGA GGG TTC GGA GT | GGA GCC ATT CTC CAT GAA TC |
| *Tnf* | TNF | GAA GTT CCC AAA TGG CCT CC | GTG AGG GTC TGG GCC ATA GA |
| *Tnfaip3* | A20 | TCT GGA GGA GTC TCA GAT GGA | GCA GGA TGT ACC TGG ACC TT |
| *Xiap* | XIAP | CGA CGC TAA TCG AGG GCC GC | TCG CGC CAA GCA CTC CAG TC |
